# Anticancer Potential of Azatetracyclic Derivatives: In Vitro Screening and Selective Cytotoxicity of Azide and Monobrominated Compounds

**DOI:** 10.3390/molecules30030702

**Published:** 2025-02-05

**Authors:** Costel Moldoveanu, Ionel I. Mangalagiu, Gheorghita Zbancioc, Ramona Danac, Gabriela Tataringa, Ana Maria Zbancioc

**Affiliations:** 1 Faculty of Chemistry, Alexandru Ioan Cuza University of Iasi, 11 Carol 1, 700506 Iasi, Romania; costel.moldoveanu@uaic.ro (C.M.); ionelm@uaic.ro (I.I.M.); 2 Institute of Interdisciplinary Research—CERNESIM Centre, Alexandru Ioan Cuza University of Iasi, 11 Carol I, 700506 Iasi, Romania; 3 Faculty of Pharmacy, University of Medicine and Pharmacy “Grigore T. Popa” Iasi, 16 University Street, 700115 Iasi, Romania; gtataringa22@yahoo.com (G.T.); ana.zbancioc@umfiasi.ro (A.M.Z.)

**Keywords:** azatetracyclic, benzo[*f*]quinoline, cycloaddition, cancer cell growth inhibition, anticancer

## Abstract

This study investigated the antiproliferative activity of three classes of benzo[*f*]pyrrolo[1,2-*a*]quinoline azatetracyclic derivatives. All compounds were screened against 60 cancer cell lines at a single dose of 10 μM. When we compared the activity of the three classes of azatetracyclic derivatives (azide, monobrominated and dibrominated), we found that the dibrominated compounds were less active, while the azides were the most active molecules. Compounds **3b** and **5a**, showing the best growth inhibition profile of all the drugs evaluated, were selected for the second stage of a full five-dose testing. According to the results of the in vitro screening, compounds **3b** and **5a** exhibit good to moderate anticancer activity (in micromolar range) against all nine cancer sub-panels, with compound **5a** being more selective than compound **3b**. Both compounds presented better activity than phenstatin on T–47D breast cancer cells, with compound **3b** also being more active on SK–MEL–28 melanoma cells, while compound **5a** was more active than phenstatin on COLO 205 colon cancer cells. As for the probable mechanism of action, the benzoquinoline derivatives could act as PI5P4Kα and PI5P4Kβ inhibitors or topoisomerase II inhibitors.

## 1. Introduction

Due to their various useful features, including complexation [1,2,3], luminescence [1,2,4], biological activities [4,5,6,7], and semiconductors [8], benzoquinolines are versatile molecules that have been used in several applications [1,2,3]. The benzoquinoline scaffold is a great option for the creation of distinctive bioactive molecules because these polycyclic compounds are also found naturally in morphine alkaloids, sterols, or hormones [9].

In addition, the combination of two or more heterocycle rings can create new classes of ligands, many of which have intriguing new features, such as anticancer activity. Analogs of nitidine and fagaronine with substituted benzo[*c*]phenanthrolines, for instance, demonstrated cytotoxic effects linked to DNA intercalation and caused arrests in the G2/M phases [10]. Our team recently revealed various fused pyrrolophenanthrolines or pyrrolobenzoquinoline that exhibited moderate antiproliferative activity [11,12].

The treatment for a variety of cancer kinds can be difficult and include stem cell transplantation, immunotherapy, hormone treatment, radiation, chemotherapy, surgery, and often, a combination of these [13,14,15]. Chemotherapy is a cancer treatment that can increase life expectancy. When the first anticancer drug was introduced into the market in 1949, the average lifespan was 46.8 years; in recent years, more than 160 anticancer drugs have been approved for clinical usage, bringing the average lifespan up to 71.4 years [16,17]. An estimated 9.7 million people died from cancer and 20 million new cases were reported in 2022. However, the drugs on the market now have a low rate of effectiveness and a host of drawbacks [18,19,20]. Even with the significant advancements in chemotherapy, cancer continues to be the primary cause of death globally. Thus, it is imperative for novel anticancer medications to be developed for use in treatment.

In light of the aforementioned considerations, our study focuses on the study of azaheterocyclic scaffolds in potential anticancer compounds [21]. Herein, we studied new hybrid compounds that include a benzo[*f*]quinoline core with possible anticancer potential.

**Scheme 1:**
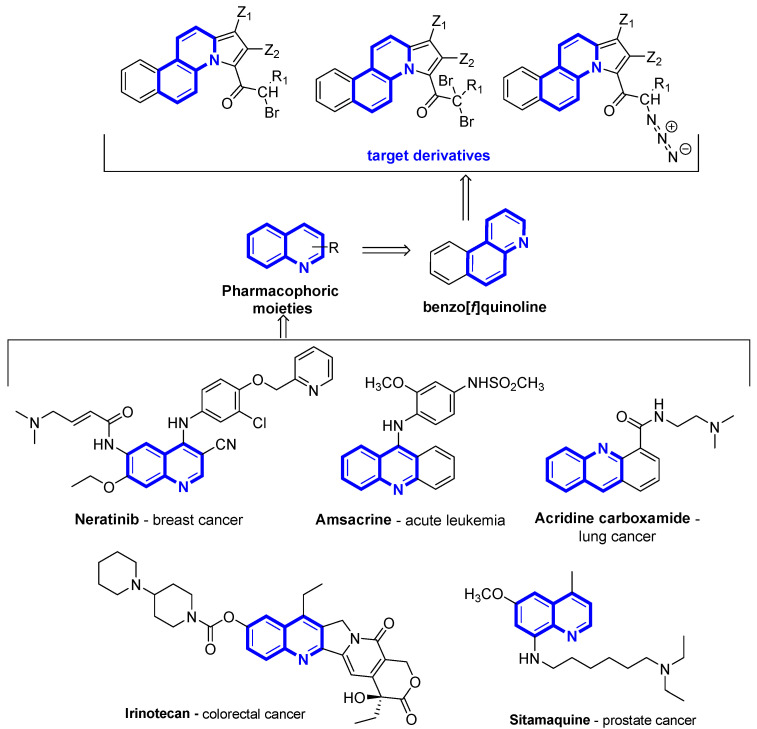
Design in the class of benzo[*f*]quinolines derivatives with anticancer activity.

In earlier studies, quinoline derivatives with polycyclic skeletons have been shown to exhibit a variety of biological activities, including anticancer properties [12,22,23]. Furthermore, quinoline- or benzo[*f*]quinoline-based substances are ATP synthase and Topo II inhibitors in terms of their mode of action [22,23,24]. Given the aforementioned factors, it seems reasonable to assume that target polycyclic benzo[*f*]quinoline functionalized derivatives, which had a similar structure to those previously tested, will function similarly in terms of their mechanism of action and antitumor activity (Scheme 1).

**Figure 1:**
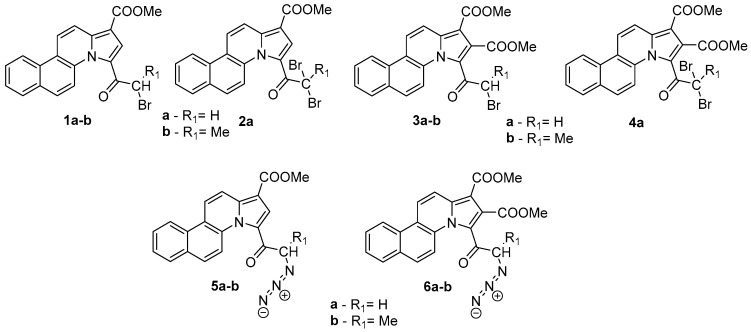
Molecular structures of benzo[*f*]quinoline derivatives.

The synthesis of target functionalized azatetracyclic derivatives was realized as presented in our previous research [25]. Figure 1 shows the molecular structures of the compounds tested as anticancer agents against different cell lines.

## 2. Results and Discussion

### 2.1. Anticancer Activity

The anticancer activity of the synthesized compounds was evaluated by the National Cancer Institute (NCI), USA, through its screening program for anticancer medications. The in vitro NCI 60 cell line screen provides a comprehensive assessment of the compounds’ efficacy against various cancer cell types, making it a valuable resource for drug development. It comprises 60 distinct human tumor cell lines that represent a range of malignancies, such as kidney, lung, colon, brain, ovary, breast, prostate, and leukemia. The screening was conducted in accordance with the NCI protocol, which is included in the accompanying documentation [26,27,28,29,30,31,32].

In the first step of the screening procedure, each selected chemical is evaluated at a single dose of 10 μM against all 60 cell lines [26]. The mean graph showing the outcomes of this screen can be examined with the COMPARE application [29]. The results are expressed and the growth, relative to the drug-free control and the initial cell count using the percentage growth inhibition, or PGI, is indicated. This method can be used to detect growth inhibition (values range from 0 to 100) and death (values less than 0). If the number was 30, for example, 70% of growth would be inhibited, and if the number was −30, 30% of the cells would be dead.

Ten synthesized compounds, including four azatetracyclic monobromoderivatives (**1a,b** and **3a,b**), two azatetracyclic dibromoderivatives (**2a** and **4a**) and four azatetracyclic azides (**5a,b** and **6a,b**), were evaluated in a first single-dose anticancer assay (at a concentration of 10^−5^ M). Table 1 contains the representative results that were obtained for all tested substances.

Compounds **3b** and **5a** were very effective in inhibiting the growth of many cancer cell lines. Compound **5a** showed the best efficacy in terms of growth inhibition against the following cell lines: CCRF–CEM (with 89% lethality); HL–60 (TB) (with 98% lethality); MOLT–4 (with 99% lethality) and RPMI–8226 (with 87% lethality) leukemia cell lines, A549/ATCC (with 40% lethality); EKVX (with 44% lethality); HOP–62 (with 20% lethality); HOP–92 (with 6% lethality); NCI–H226 (with 2% lethality); NCI–H23 (with 2% lethality); NCI–H322M (with 15% lethality) and NCI–460 (with 88% lethality) non-small cell lung cancer lines, COLO 205 (with 44% lethality) and HCC–2998 (with 9% lethality) colon cancer cell lines, SF–295 (with 15% lethality); SF–539 (with 1% lethality) and SNB–75 (with 8% lethality) CNS cancer cell lines, SK–OV–3 (with 61% lethality) ovarian cancer cell line, A498 (with 47% lethality) and ACHN (with 7% lethality) renal cancer cell lines, DU–145 (with 2% lethality) prostate cancer cell line and HS 578T (with 19% lethality) and MDA–MB–468 (with 52% lethality) breast cancer cell lines.

Additionally, compound **3b** demonstrated cytotoxic activity against the following cell lines: CCRF–CEM (with 4% lethality) leukemia cell line, NCI–H522 (with 55% lethality) non-small cell lung cancer line and LOX IMVI (with 5% lethality) melanoma cell line.

Regarding the leukemia cell line, compounds **5b** and **6b** show significant anticancer activity with great lethality against three (HL–60 (TB); MOLT–4; SR) and four (CCRF–CEM; MOLT–4; RPMI–8226; SR) cancer cell lines, respectively.

The analysis of the three azatetracyclic cycloadduct classes revealed a clear cancer cells inhibition hierarchy: azides demonstrated the highest activity, followed by monobrominated derivatives, while dibrominated compounds showed the lowest growth inhibition levels.

**Figure 2:**
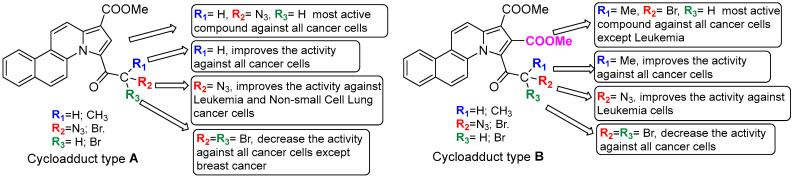
SAR diagram in the series of benzo[*f*]pyrrolo[1,2-*a*]quinoline azatetracyclic derivatives. (**A**—cycloadduct from methyl propiolate; **B**—cycloadduct from dimethyl acetylenedicarboxylate).

Considering the structure–activity relationship (SAR), a number of preliminary conclusions can be drawn (Figure 2).

Among the cycloadducts of type **A**, the unsubstituted azide **5a** (R_1_ = H) exhibited the best inhibitory potential. In general, derivatives with R_1_ = H showed superior anticancer activity across most cell lines compared to compounds with R_1_ = Me. The presence of an azido group (R_2_ = N_3_) enhanced activity against leukemia and non-small cell lung cancer cells specifically. Dibrominated compounds exhibited the lowest potency against all cancer types, except breast cancer.

For type **B** cycloadducts, the methyl-substituted monobrominated derivative **3b** (R_1_ = Me) demonstrated the highest activity across all cancer types except leukemia. In contrast to type **A**, compounds bearing R_1_ = Me provided better inhibitory properties compared to the ones with R_1_ = H. The azido substitution (R_2_ = N_3_) selectively enhanced the activity against leukemia cells. Dibrominated derivatives showed the lowest activity across all cancer cell lines, which was a similar behavior as in the case of type **A** compounds.

Based on their specific anticancer profiles, compounds **3b** and **5a** were selected for comprehensive five-dose evaluation studies. Phenstatin served as a reference compound under identical testing conditions. Table 2 shows a selection of the GI_50_ values.

According to the results of the in vitro screening, compound **3b** exhibits good to moderate anticancer activity against the cancer cell lines from all nine sub-panels, with GI_50_ values ranging from 2.79 to 39.9 µM (the GI_50_ value was no longer determined if it was higher than 42.2 µM). Specifically, compound **3b** had the best GI_50_ and TGI (the concentration that results in total growth inhibition) values against RXF 393 renal cancer cells (2.79 µM and 36.7 µM, respectively). Additionally, promising GI_50_ values were found against all leukemia cell lines, on three breast cancer cells (MCF7; MDA-MB-231/ATCC; BT-549), on two melanoma cells (LOX IMVI; MALME–3M), and on two colon cancer cells (HCT–15; SW-620).

Compound **5a** exhibited anticancer activity on fewer cell lines, compared to compound **3b**, on the 60 cancer cell lines from all nine sub-panels, with GI_50_ values ranging from 1.41 to 29.8 µM. In particular, compound **5a** had the best GI_50_ and TGI values against A549/ATCC non-small cell lung cancer (1.41 µM and 6.76 µM, respectively).

When comparing the anticancer activity of the two compounds tested with the control phenstatin, both compounds showed better activity on T-47D breast cancer cells; compound **3b** was more active than phenstatin on SK-MEL-28 melanoma cells, while compound **5a** was more active than phenstatin on COLO 205 colon cancer cells.

In order to determine a possible mode of action of compounds **3b** and **5a**, we used in silico platform-approved and Investigational Oncology Agents COMPARE (IOA COMPARE) of NCI [29]. This NCI platform is built on top of the NCI 60 screening data for a set of 183 FDA-approved oncology drugs and 820 investigational agents. The similarity of pattern to that of the analyzed compound is expressed quantitatively as a Pearson correlation coefficient. The results obtained with the COMPARE algorithm indicate that compounds high in this ranking (correlation coefficient > 0.5) may possess a mechanism of action similar to that of the seed compound. When analyzing compound **3b** with IOA COMPARE, we found a best-fitting profile (correlation coefficient = 0.68) with (R)-8-cyclopentyl-7-(cyclopentylmethyl)-2-((3,5-dichloro-4-hydroxyphenyl)amino)-5-methyl-7,8-dihydropteridin-6(5H)-one, known as CC260, a highly potent and selective noncovalent dual inhibitor for both phosphatidylinositol 5-phosphate 4-kinases PIP4Kα and PIP4Kβ. In the case of compound **5a**, the best-fitting profile was obtained for (S)-4,4′-(propane-1,2-diyl)bis(piperazine-2,6-dione, Dexrazoxane), an FDA-approved cardioprotective drug used to ameliorate cardiac toxicity seen in anthracycline-based (e.g., doxorubicin, daunorubicin, epirubicin) chemotherapy recipients for cancer by fusing with free and bound iron. Dexrazoxane also acts as a DNA topoisomerase II inhibitor, which happens to be the same target of the DNA topoisomerase II anticancer agent (e.g., the anthracyclines), antagonizing the formation of the topoisomerase II cleavage complex and also rapidly degrading topoisomerase II beta [33].

The mean graphs comparison of the two compounds **3b** and **5a** are presented in Figures S13 and S14, respectively (Supplementary Materials) [34].

### 2.2. In Silico ADME and Toxicity Profile

Given the promising anticancer activity shown in our results, it becomes essential to further evaluate the drug candidates’ pharmacokinetic and toxicological profile. Thus, we performed an in silico ADMET (absorption, distribution, metabolism, excretion, and toxicity) study to theoretically assess the potential of compounds **3b** and **5a** as therapeutic agents. The predicted parameters, including molecular properties, pharmacokinetics, drug-likeness, and medicinal chemistry, are summarized in Table 3.

Compounds **3b** and **5a** exhibit favorable drug-like properties, fully complying with both Lipinski’s rule of five and Veber’s guidelines. Specifically, these compounds meet all the criteria for molecular properties: they have no violations of Lipinski’s rule, maintain fewer than 10 rotatable bonds, and possess a topological polar surface area below 140 Å^2^.

Compound **3b** and **5a** exhibit limited aqueous solubility, with a Log S (ESOL) value of ~−6.00. While the compounds present three Brenk structural alerts, these structural features do not necessarily preclude their potential pharmaceutical utility and may, in fact, contribute to their unique molecular characteristics.

Derivatives **3b** and **5a** exhibit promising pharmacokinetic characteristics, as detailed in Table 3. The compounds are predicted to have high gastrointestinal absorption, though they cannot permeate the blood–brain barrier (BBB). Furthermore, both compounds are not anticipated to act as a P-glycoprotein (P-gp) substrate, which can be beneficial for targeted drug delivery.

**Figure 3:**
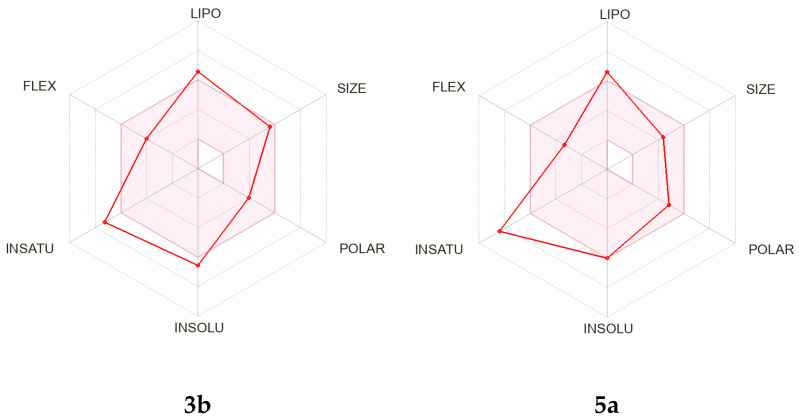
SwissADME bioavailability chart of compounds **3b** and **5a**.

The charts derived from the Swiss ADME QSAR web tool, depicting the oral bioavailability predictions for compounds **3b** and **5a**, are illustrated in Figure 3.

The chart evaluates six key molecular parameters for drug-likeness: lipophilicity (LIPO), molecular size, polarity (POLAR), insolubility (INSOLU), unsaturation (INSATU), and molecular flexibility (FLEX). The chart represents these parameters with a red line. If the red line is integrated into the pink area, the molecule is considered drug-like. Compounds 3b and **5a** demonstrated partial matches, meeting three (compound **3b**) or four (compound **5a**) of the six parameters while both exhibiting violations in two areas: INSATU (the ratio of sp^3^-hybridized carbon atoms to the total number of carbon atoms) and LIPO (with an XLOGP3 value falling outside the acceptable range of −0.7 to +5.0). Despite these minor deviations, the estimated results, together, suggest a promising pharmacokinetic and drug-likeness profile for both compounds.

The compound’s toxicity profile was analyzed using in silico predictions that generate probability scores for both active (Pa) and inactive (Pi) states across different biological targets (Table 4).

As shown in Table 4, compound **5a** demonstrated the predicted cytotoxicity (defined by Pa > Pi and Pa > 0.3) against multiple cancer cell lines, notably against all six lines that were experimentally tested in the NCI panel: SF-539 glioblastoma, UACC-62 melanoma, SN12C renal carcinoma, HOP-62 non-small cell lung carcinoma, OVCAR-3 ovarian carcinoma and T47D breast carcinoma (Table 3). The predictions for compound **5b** indicated potential cytotoxic activity against three cancer cell lines: MOLT-3, CCRF-CEM and NCI-H187. In this case, our experimental testing also confirmed the cytotoxicity against CCRF-CEM leukemia cells (Table 3), providing a validation of the computational predictions. Importantly, the prediction algorithm did not indicate any significant toxicity against normal human cell lines, suggesting potential selective anticancer activity for both compounds **3b** and **5a**. However, further experimental validation would be needed to confirm this selectivity.

## 3. Experimental Section

### 3.1. Synthetic Procedure

All ten tested compounds were synthesized according to our procedure previously reported in the literature [25].

### 3.2. Biological Testing

#### Cell Proliferation Assay

The in vitro biological studies were conducted by the National Cancer Institute (NCI, Bethesda, MD, USA) as part of the Developmental Therapeutics Program (DTP).

The Developmental Therapeutics Program (DTP) at the NCI has successfully guided late-stage preclinical therapeutics through the critical stages of development for more than 50 years. As a result, this initiative has been responsible for the discovery and development of more than 70% of the anticancer drugs used in modern therapy [30].

This screen works with 60 different human tumor cell lines representing leukemia, melanoma and malignancies of the ovary, breast, prostate, kidney, colon, and brain [31]. The goal is to prioritize synthetic chemicals or natural product samples that selectively inhibit the growth of or kill specific tumor cell lines for further evaluation. This screen is unique in that the biological response pattern generated by the complex dose–response of a given compound in 60 cell lines can be used by the COMPARE program in pattern recognition algorithms [29].

The evaluation of all compounds against the 60 cell lines at a single dose of 10^−5^ M is the first screening stage.

The standard NCI/DTP in vitro cancer screening methodology was used [32].

All 60 human malignant tumor cell lines were cultured in RPMI (Roswell Park Memorial Institute) 1640 medium containing 5% fetal bovine serum and 2 × 10^−3^ M L-glutamine. For a standard screening test, 100 μL of cancer cells are injected into 96-well microtiter plates (at plating densities ranging from 5000 to 40,000 cells/well, depending on the cell division rate of certain cell lines). After cell inoculation, the microtiter plates are incubated for 24 h under specified conditions before the addition of experimental drugs: 37 °C, 5% CO_2_, 95% air and 100% relative humidity. Two plates for each cell line are fixed in situ with TCA after 24 h to obtain measurements of each cell population at the time of test substance (Tz) addition. Prior to use, the test chemicals are frozen after being solubilized in DMSO (dimethylsulphoxide) at 400 times the intended final maximum test concentration. After the test substance has been added, a portion of the frozen concentrate is thawed, diluted two-fold and then mixed with a medium containing 50 µg/mL gentamicin. The required final drug concentration (10^−5^ M) is achieved by adding 100 µL of the test substance to the microtiter wells already containing 100 µL of medium. After the addition of the test chemical, the microtiter plates are incubated for a further 48 h under the conditions described above. The addition of cold TCA completes the test in the case of adherent cells. Cancer cells are fixed by the addition of 50 µL of cold 50 % (*w*/*v*) TCA (final concentration, 10% TCA). This is followed by incubation at 4 °C for 60 min. The plates are air dried after five rinses with tap water and the supernatant is discarded. After drying, 100 µL of a fluorescent dye solution (sulforhodamine B) is added to each well. The plates are then incubated at 25 °C for 10 min. The plates are washed five times with a 1% acetic acid solution to remove unbound fluorophore and then air dried. A 10 × 10^−3^ M Trisma base is used to solubilize the bound fluorophore and the absorbance is measured at 515 nm using an automated plate reader. The procedure is the same for the suspension cells, except that settled cells at the bottom of the wells are fixed by adding 50 µL of TCA 80% solution (final concentration, 16% TCA) to the test.

Calculate the percentage growth inhibition (PGI) from the seven absorbance readings as follows:

[(Ti − Tz)/(C − Tz)] × 100 (for concentrations for which Ti >/= Tz);

[(Ti − Tz)/Tz] × 100 (for concentrations for which Ti < Tz).

where Ti = test growth in the presence of the drug at the concentration level, C = control growth, and Tz = time zero.

Three dose–response parameters are derived for each chemical tested.

### 3.3. In Silico ADMET Predictions

The ADME in silico evaluation of the compounds **3b** and **5a** was performed using the SwissADME web tool (http://swissadme.ch/index.php (accessed on 10 December 2024)) in terms of molecular properties, pharmacokinetics, drug-likeness, and medicinal chemistry [35].

The in silico toxicological evaluation for the most active compounds **3b** and **5a** was performed using the webservice Cell Line Cytotoxicity Predictor (CLC-Pred) (https://www.way2drug.com/clc-pred/ (accessed on 13 January 2025)), which screens for in silico cytotoxicity on a panel of 278 tumor cells and 27 normal human cell lines from different tissues [36].

## 4. Conclusions

In conclusion, we report herein the anticancer potential of three classes of azatetracyclic derivatives (monobrominated, dibrominated and azide). Testing using a single-dose (10^−5^ M) experiment showed azide **5a** being the most active compound, showing very good lethality against the majority of the cell lines tested (with a lethality in the range of 2–99%). Very good anticancer activity was also demonstrated by the monobrominated azatetracyclic derivative **3b**, but also by all other azide derivatives **5b** and **6a**–**b** that showed significant selective lethality for the leukemia and non-small cell lung cancer line. In addition, compound **3b** showed selective growth inhibitory activity against the LOX IMVI melanoma cell line.

An SAR analysis revealed distinct inhibitory patterns for cycloadducts **A** and **B**. In type **A** compounds, cancer cell growth inhibition was optimized by an unsubstituted position (R_1_ = H) and azido substitution (R_2_ = N_3_), with compound **5a** showing the highest potency. Conversely, type **B** compounds benefited from methyl substitution (R_1_ = Me), exemplified by the potent monobrominated derivative **3b**. Notably, dibromination consistently reduced the activity across both series, while azido substitution enhanced the activity against specific cancer types, particularly leukemia.

The five-dose testing of compounds **3b** and **5a** shows anticancer activity in the micromolar range across all nine cancer sub-panels, with compound **5a** proving more selective for several cancer cell lines than compound **3b**. Both compounds also showed better activity on T-47D breast cancer cells than the control phenstatin. Compound **3b** was more active than phenstatin on SK–MEL–28 melanoma cells, while compound **5a** was more active than phenstatin on COLO 205 colon cancer cells. The IOA COMPARE NCI’s platform suggested that the anticancer activity of the benzoquinolines derivative could be achieved by the inhibition of PI5P4K kinase for compound **3b**, or by topoisomerase II inhibition in the case of compound **5a**. The in silico ADMET showed interesting pharmacokinetic properties and toxicity profiles. Considering their potential, compounds **3b** and **5a** could be further optimized.

## Figures and Tables

**Table 1:** Single-dose (10^−5^ M) assay results of the functionalized benzo[*f*]quinolinium derivatives against 60 NCI human cancer cell lines, expressed as the percentage growth inhibition (PGI%).

Cell Type	Compound/Percentage Growth Inhibition (PGI%) ^a^									
	1a	1b	2a	3a	3b	4a	5a	5b	6a	6b
Leukemia										
CCRF–CEM	**87**	65	65	28	**100 (4)** ^b^	42	**100 (89)** ^b^	59	68	**100 (19)** ^b^
HL–60 (TB)	51	18	24	7	61	10	**100 (98)** ^b^	**100 (38)** ^b^	33	66
K–562	58	33	48	36	**96**	44	0	52	35	66
MOLT–4	**94**	68	78	45	**99**	35	**100 (99)** ^b^	**100 (96)** ^b^	**88**	**100 (93)** ^b^
RPMI–8226	62	29	46	31	**97**	29	**100 (87)** ^b^	**84**	55	**100 (30)** ^b^
SR	**98**	**87**	**87**	44	**97**	65	-	**100 (59)** ^b^	**100 (6)** ^b^	**100 (97)** ^b^
Non–small Cell Lung Cancer										
A549/ATCC	69	46	60	8	50	0	**100 (40)** ^b^	51	6	0
EKVX	40	14	57	1	14	0	**100 (44)** ^b^	**90**	**100 (1)** ^b^	36
HOP–62	52	25	44	13	33	0	**100 (20)** ^b^	-	-	-
HOP–92	47	26	46	0	47	0	**100 (6)** ^b^	56	15	27
NCI–H226	47	22	36	0	39	0	**100 (2)** ^b^	**100 (5)** ^b^	47	**80**
NCI–H23	49	17	31	7	67	2	**100 (2)** ^b^	55	18	35
NCI–H322M	18	6	11	4	17	0	**100 (15)** ^b^	0	3	21
NCI–460	61	60	82	0	**83**	0	**100 (88)** ^b^	77	17	49
NCI–H522	41	28	40	27	**100 (55)** ^b^	10	**93**	46	41	**100 (44)** ^b^
Colon Cancer										
COLO 205	62	0	45	0	57	0	**100 (44)** ^b^	35	0	10
HCC–2998	38	0	57	0	33	0	**100 (9)** ^b^	19	0	5
HCT–116	65	23	51	5	**86**	9	77	47	19	49
HCT–15	61	39	66	31	**91**	18	**92**	32	5	19
HT29	56	16	37	14	77	5	67	20	13	24
SW–620	45	34	55	6	**91**	29	64	61	0	34
CNS Cancer										
SF–268	26	4	32	0	31	1	77	76	16	47
SF–295	63	22	47	0	22	0	**100 (15)** ^b^	-	-	-
SF–539	57	27	46	2	**83**	3	**100 (1)** ^b^	45	10	36
SNB–19	45	17	42	15	48	0	72	53	19	30
SNB–75	10	2	34	0	6	0	**100 (8)** ^b^	72	50	49
U251	61	50	56	30	77	13	75	-	-	-
Melanoma										
LOX IMVI	69	58	59	56	**100 (5)** ^b^	28	71	52	36	64
MDA–MB–435	26	0	21	17	**90**	4	39	0	0	10
SK–MEL–2	0	0	0	10	74	0	61	20	24	**87**
Ovarian Cancer										
OVCAR–3	0	0	24	4	84	1	**92**	34	23	58
OVCAR–8	69	44	62	20	**85**	16	74	65	26	44
SK–OV–3	38	9	25	0	19	0	**100 (61)** ^b^	25	17	4
Renal Cancer										
786–0	48	12	36	3	59	0	**81**	55	18	28
A498	0	0	0	0	13	0	**100 (47)** ^b^	53	18	18
ACHN	83	31	64	0	57	0	**100 (7)** ^b^	65	31	31
CAKI–1	47	30	42	3	29	4	77	52	23	53
RXF 393	34	0	0	0	68	0	**89**	-	-	-
SN12C	49	33	47	4	65	0	**93**	70	24	22
TK–10	16	12	35	0	42	0	**99**	61	33	43
Prostate Cancer										
DU–145	31	35	48	0	29	0	**100 (2)** ^b^	38	10	14
Breast Cancer										
MCF7	**94**	61	**95**	30	**94**	38	**87**	63	58	52
HS 578T	40	31	56	0	35	0	**100 (19)** ^b^	**100 (12)** ^b^	61	76
T–47D	43	24	**99**	40	64	23	-	**99**	48	42
MDA–MB–468	52	0	**100 (8)** ^b^	15	50	0	**100 (52)** ^b^	-	-	-

^a^ values are reported as a one-dose assay; the percentage growth inhibition (PGI) is growth relative to the no-drug control, and relative to the number of cells at time zero; ^b^ cytotoxic effect; percentage lethality is shown in brackets; bold highlighted values are the best in the series.

**Table 2:** Results of the five-dose in vitro human cancer cell growth inhibition ^a^ for compounds **3b** and **5a** and the control, phenstatin.

Cell Type	GI_50_ (μM) ^b^		
	Compound 3b	Compound 5a	Phenstatin
Leukemia			
CCRF–CEM	**3.92**	24.7	0.034
HL–60 (TB)	**5.39**	12.7	0.011
K–562	**6.48**	>42.2	-
MOLT–4	**3.30**	**6.58**	-
RPMI–8226	**7.43**	13.6	-
SR	**5.87**	**8.32**	<0.010
Non-small Cell Lung Cancer			
A549/ATCC	>42.2	**1.41**	-
EKVX	>42.2	**8.77**	-
HOP–62	33.1	>42.2	0.073
HOP–92	15.2	>42.2	-
NCI–H226	25.6	>42.2	-
NCI–H23	12.9	>42.2	-
NCI–H322M	>42.2	12.8	-
NCI–460	17.8	**3.07**	0.033
NCI–H522	**3.07**	>42.2	0.027
Colon Cancer			
COLO 205	38.8	**4.18**	4.86
HCC–2998	39.9	**6.73**	-
HCT–116	11.0	>42.2	0.038
HCT–15	**6.17**	>42.2	<0.010
HT29	20.6	>42.2	2.95
KM12	>42.2	>42.2	<0.010
SW–620	**7.26**	>42.2	<0.010
CNS Cancer			
SF–268	34.4	>42.2	-
SF–295	28.3	>42.2	0.367
SF–539	10.9	>42.2	0.011
SNB–19	>42.2	>42.2	-
SNB–75	24.7	>42.2	<0.010
U251	18.3	>42.2	0.043
Melanoma			
LOX IMVI	**4.35**	>42.2	0.013
MALME–3M	**6.10**	>42.2	-
M14	31.3	>42.2	<0.010
MDA–MB–435	16.1	>42.2	<0.010
SK–MEL–2	17.9	>42.2	0.520
SK–MEL–28	25.7	>42.2	65.20
SK–MEL–5	13.0	>42.2	0.040
UACC–257	>42.2	>42.2	-
UACC–62	11.9	>42.2	0.448
Ovarian Cancer			
IGROV1	33.2	>42.2	0.18
OVCAR–3	16.9	>42.2	0.021
OVCAR–4	25.6	>42.2	-
OVCAR–5	22.5	>42.2	-
OVCAR–8	14.9	>42.2	0.042
NCI/ADR–RES	17.7	>42.2	0.012
SK–OV–3	>42.2	11.9	-
Renal Cancer			
786–0	21.1	>42.2	0.905
A498	>42.2	**5.71**	2.28
ACHN	22.2	>42.2	0.042
CAKI–1	21.2	27.8	-
RXF 393	**2.79**	>42.2	0.016
SN12C	17.2	29.8	-
TK–10	>42.2	>42.2	-
UO–31	24.9	>42.2	0.074
Prostate cancer			
PC–3	17.8	>42.2	0.045
DU–145	>42.2	25.3	0.039
Breast cancer			
MCF7	**4.58**	24.5	0.033
MDA–MB–231/ATCC	**4.72**	>42.2	0.029
HS 578T	18.8	>42.2	0.031
BT–549	**6.18**	>42.2	0.034
T–47D	11.5	18.1	30.4
MDA–MB–468	13.2	**7.25**	2.71

^a^ the data were obtained from NCI’s in vitro 60 cell five-dose screening. ^b^ GI_50_—the molar concentration of the tested compound causing the 50% growth inhibition of tumor cells. The GI_50_ value was no longer determined if it was more than 42.2 µM. Bold highlighted values are the best in the series.

**Table 3:** In silico prediction of ADME parameters for compounds **3b** and **5a**.

ADME Parameter	3b	5a
***Physicochemical Properties***		
Molecular weight	468.30 g/mol	358.35 g/mol
Log *P*_o/w_ (MLOGP)	3.24	1.94
Number of H-bond acceptors	5	6
Number of H-bond donors	0	0
Number of rotatable bonds	6	5
TPSA	74.08 Å^2^	97.53 Å^2^
***Pharmacokinetics***		
Gastrointestinal (GI) absorption	high	high
Blood–brain barrier (BBB) permeant	no	no
P-gp substrate	no	no
***Drug-likeness***		
Log S (ESOL)	−6.55	−6.02
Water solubility class	poorly soluble	poorly soluble
Lipinski rule	no violation	no violation
Veber rule	no violation	no violation
Bioavailability	0.55	0.55
***Medicinal Chemistry***		
PAINS alerts	0	1 alert: azo
Brenk alerts	3 alerts: alkyl_halide, more than 2 esters, polycyclic aromatic hydrocarbon	4 alerts: azido_group, diazo_group, polycyclic aromatic hydrocarbon, quaternary nitrogen
Synthetic accessibility	3.65	2.54

**Table 4:** Results of the prediction for cytotoxicity of compounds **3b** and **5a**.

**3b**			
**P_a_**	**P_i_**	**Cell Line**	**Cell Type**
0.567	0.001	MOLT-3	T-lymphoblastic leukemia
0.381	0.033	CCRF-CEM	Childhood T acute lymphoblastic leukemia
0.402	0.069	NCI-H187	Small cell lung carcinoma
**5a**			
**P_a_**	**P_i_**	**Cell Line**	**Cell Type**
0.437	0.019	SF-539	Glioblastoma
0.433	0.021	UACC-62	Melanoma
0.419	0.028	SN12C	Renal carcinoma
0.419	0.028	HOP-62	Non-small cell lung carcinoma
0.379	0.036	OVCAR-3	Ovarian adenocarcinoma
0.318	0.060	T47D	Breast carcinoma

## Data Availability

Data are contained within the article and Supplementary Materials.

## Supplementary Materials

The following supporting information can be downloaded at: https://www.mdpi.com/article/10.3390/molecules30030702/s1, Figure S1: Anticancer activity (single-dose (10^−5^ M) assay) of the azatetracyclic derivative **1a**; Figure S2: Anticancer activity (single-dose (10^−5^ M) assay) of the azatetracyclic derivative **1b**; Figure S3: Anticancer activity (single-dose (10^−5^ M) assay) of the azatetracyclic derivative **2a**; Figure S4: Anticancer activity (single-dose (10^−5^ M) assay) of the azatetracyclic derivative **3a**; Figure S5: Anticancer activity (single-dose (10^−5^ M) assay) of the azatetracyclic derivative **3b**; Figure S6: Anticancer activity (single-dose (10^−5^ M) assay) of the azatetracyclic derivative **4a**; Figure S7: Anticancer activity (single-dose (10^−5^ M) assay) of the azatetracyclic derivative **5a**; Figure S8: Anticancer activity (single-dose (10^−5^ M) assay) of the azatetracyclic derivative **5b**; Figure S9: Anticancer activity (single-dose (10^−5^ M) assay) of the azatetracyclic derivative **6a**; Figure S10: Anticancer activity (single-dose (10^−5^ M) assay) of the azatetracyclic derivative **6b**; Figure S11: Anticancer activity (five-dose assay) of the azatetracyclic derivative **3b**; Figure S12: Anticancer activity (five-dose assay) of the azatetracyclic derivative **5a**; Figure S13: Comparison of mean graphs of compounds **3b** and **CC260**; Figure S14: Comparison of mean graphs of compounds **5a** and **dexrazoxane**. Full single-dose and five-dose screen results of the tested compounds and the COMPARE mean graphs of compounds **3b** and **5a** are provided in the supporting information.
