# Supplementary material for: Anticancer Potential of Azatetracyclic Derivatives: In Vitro Screening and Selective Cytotoxicity of Azide and Monobrominated Compounds
Source: Molecules. 2025 Feb 5;30(3):702. doi: 10.3390/molecules30030702 (PMC11820345; doi:10.3390/molecules30030702)
Supplement: Supplementary file 1 [file molecules-30-00702-s001.zip › molecules-3415042-supplementary.pdf]

Supporting Information for:

Anticancer Potential of Azatetracyclic Derivatives: In Vitro Screening and Selective Cytotoxicity of Azide and Monobrominated Compounds

Costel Moldoveanu [1], Ionel I. Mangalagiu [1,2], Gheorghita Zbancioc [1,*], Ramona Danac [1,*], Gabriela Tataringa [3] and Ana Maria Zbancioc [3]

[1]Faculty of Chemistry, Alexandru Ioan Cuza University of Iasi, 11 Carol 1st Bvd, Iasi 700506, Romania
[2]Institute of Interdisciplinary Research- CERNESIM Centre, Alexandru Ioan Cuza University of Iasi, 11 Carol I, Iasi 700506, Romania
[3]Faculty of Pharmacy, University of Medicine and Pharmacy "Grigore T. Popa" Iasi, 16 University Street, Iasi 700115, Romania

*Corresponding authors
rdanac@uaic.ro (RD)
gheorghita.zbancioc@uaic.ro (GZ)

Contents

1. Anticancer activity single dose (10^{-5} M) assay 2

2. Anticancer activity five-dose assay 12

3. Comparison of the anticancer activity of compounds 3b/ CC260 and 5a/ dexrazoxane respectively 14

1. Anticancer activity single dose (10^{-5} M) assay

Developmental Therapeutics Program One Dose Mean Graph	NSC: D-834420 / 1	Conc: 1.00E-5 Molar	Test Date: Feb 14, 2022
	Experiment ID: 2202OS68		Report Date: May 16, 2022

Panel/Cell Line	Growth Percent
**Leukemia**	
CCRF-CEM	13.99
HL-60(TB)	49.30
K-562	42.98
MOLT-4	6.44
RPMI-8226	38.72
SR	2.25
**Non-Small Cell Lung Cancer**	
A549/ATCC	31.28
EKVX	60.06
HOP-62	48.91
HOP-92	53.16
NCI-H226	53.45
NCI-H23	51.38
NCI-H322M	82.83
NCI-H460	39.39
NCI-H522	59.13
**Colon Cancer**	
COLO 205	38.73
HCC-2998	62.45
HCT-116	35.56
HCT-15	39.26
HT29	44.61
KM12	83.96
SW-620	55.44
**CNS Cancer**	
SF-268	74.71
SF-295	37.17
SF-539	43.55
SNB-19	55.46
SNB-75	90.17
U251	39.40
**Melanoma**	
LOX IMVI	31.87
MALME-3M	51.15
M14	69.47
MDA-MB-435	74.91
SK-MEL-2	113.12
SK-MEL-28	92.29
SK-MEL-5	53.39
UACC-257	82.58
UACC-62	51.22
**Ovarian Cancer**	
IGROV1	33.50
OVCAR-3	102.77
OVCAR-4	101.45
OVCAR-5	72.80
OVCAR-8	31.27
NCI/ADR-RES	57.37
SK-OV-3	62.04
**Renal Cancer**	
786-0	52.11
A498	100.96
ACHN	17.44
CAKI-1	53.97
RXF 393	66.96
SN12C	51.12
TK-10	84.35
UO-31	24.32
**Prostate Cancer**	
PC-3	53.08
DU-145	69.30
**Breast Cancer**	
MCF7	6.75
MDA-MB-231/ATCC	69.76
HS 578T	60.96
T-47D	57.66
MDA-MB-468	48.83
Mean	54.79
Delta	52.54
Range	110.87

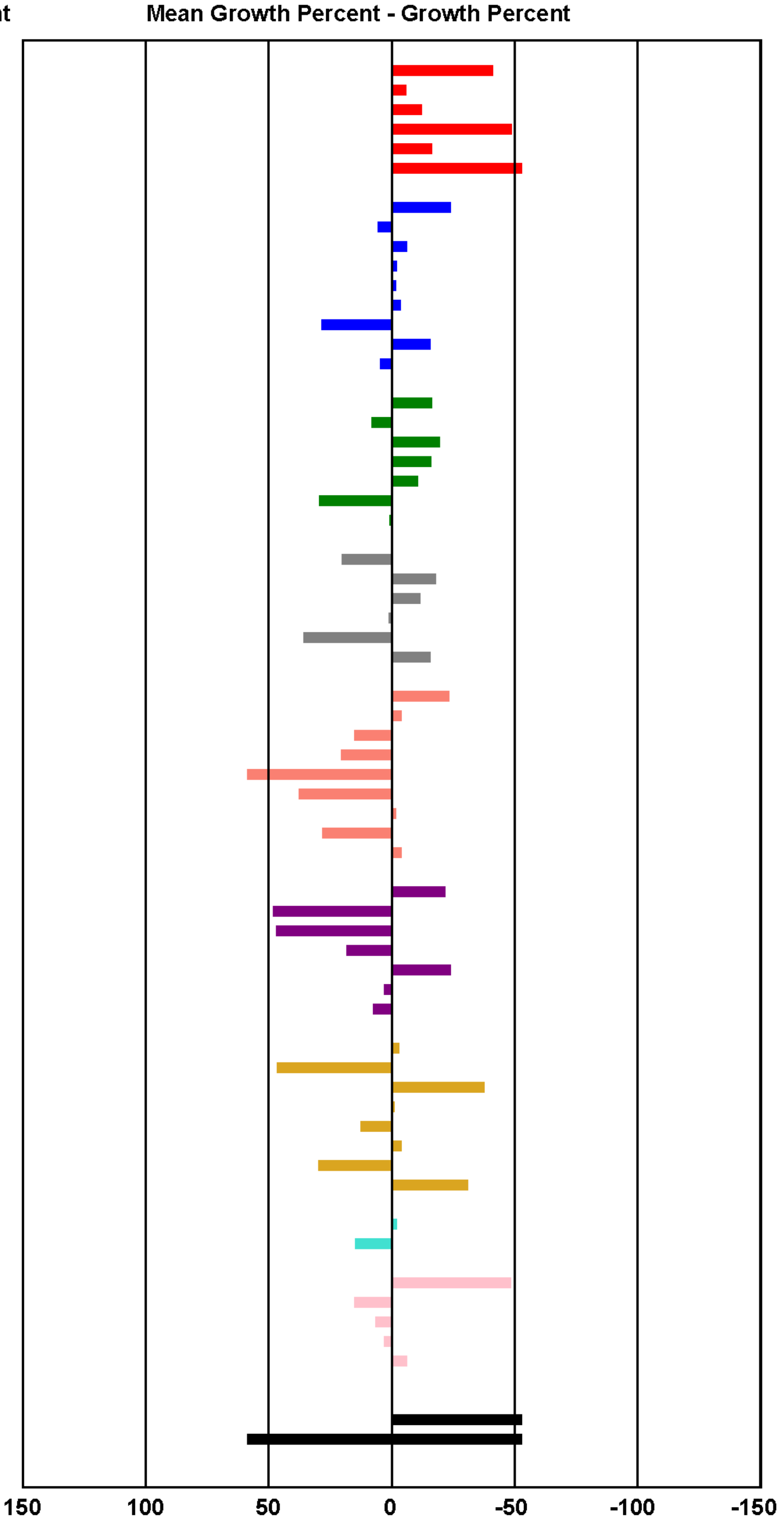

S1 Fig. Anticancer activity (single-dose (10^{-5} M) assay) of the azatetracyclic derivative **1a**.

Developmental Therapeutics Program	NSC: D-834422 / 1	Conc: 1.00E-5 Molar	Test Date: Feb 14, 2022
One Dose Mean Graph	Experiment ID: 2202OS68		Report Date: May 16, 2022

Panel/Cell Line	Growth Percent
Leukemia	
CCRF-CEM	35.82
HL-60(TB)	82.90
K-562	67.95
MOLT-4	32.75
RPMI-8226	71.17
SR	13.36
Non-Small Cell Lung Cancer	
A549/ATCC	54.86
EKVX	86.96
HOP-62	75.02
HOP-92	74.06
NCI-H226	78.46
NCI-H23	83.81
NCI-H322M	94.79
NCI-H460	40.66
NCI-H522	72.57
Colon Cancer	
COLO 205	104.15
HCC-2998	107.65
HCT-116	77.37
HCT-15	61.01
HT29	84.85
KM12	96.29
SW-620	66.55
CNS Cancer	
SF-268	96.53
SF-295	78.56
SF-539	73.08
SNB-19	83.49
SNB-75	98.98
U251	50.78
Melanoma	
LOX IMVI	32.01
MALME-3M	80.04
M14	83.29
MDA-MB-435	101.37
SK-MEL-2	115.66
SK-MEL-28	109.50
SK-MEL-5	87.98
UACC-257	91.82
UACC-62	64.55
Ovarian Cancer	
IGROV1	48.26
OVCAR-3	104.15
OVCAR-4	113.37
OVCAR-5	103.77
OVCAR-8	56.08
NCI/ADR-RES	88.53
SK-OV-3	91.50
Renal Cancer	
786-0	88.92
A498	136.75
ACHN	69.58
CAKI-1	70.87
RXF 393	111.55
SN12C	67.41
TK-10	88.66
UO-31	49.98
Prostate Cancer	
PC-3	79.52
DU-145	65.45
Breast Cancer	
MCF7	39.24
MDA-MB-231/ATCC	78.84
HS 578T	69.99
T-47D	76.18
MDA-MB-468	101.69
Mean	78.15
Delta	64.79
Range	123.39

Mean Growth Percent - Growth Percent

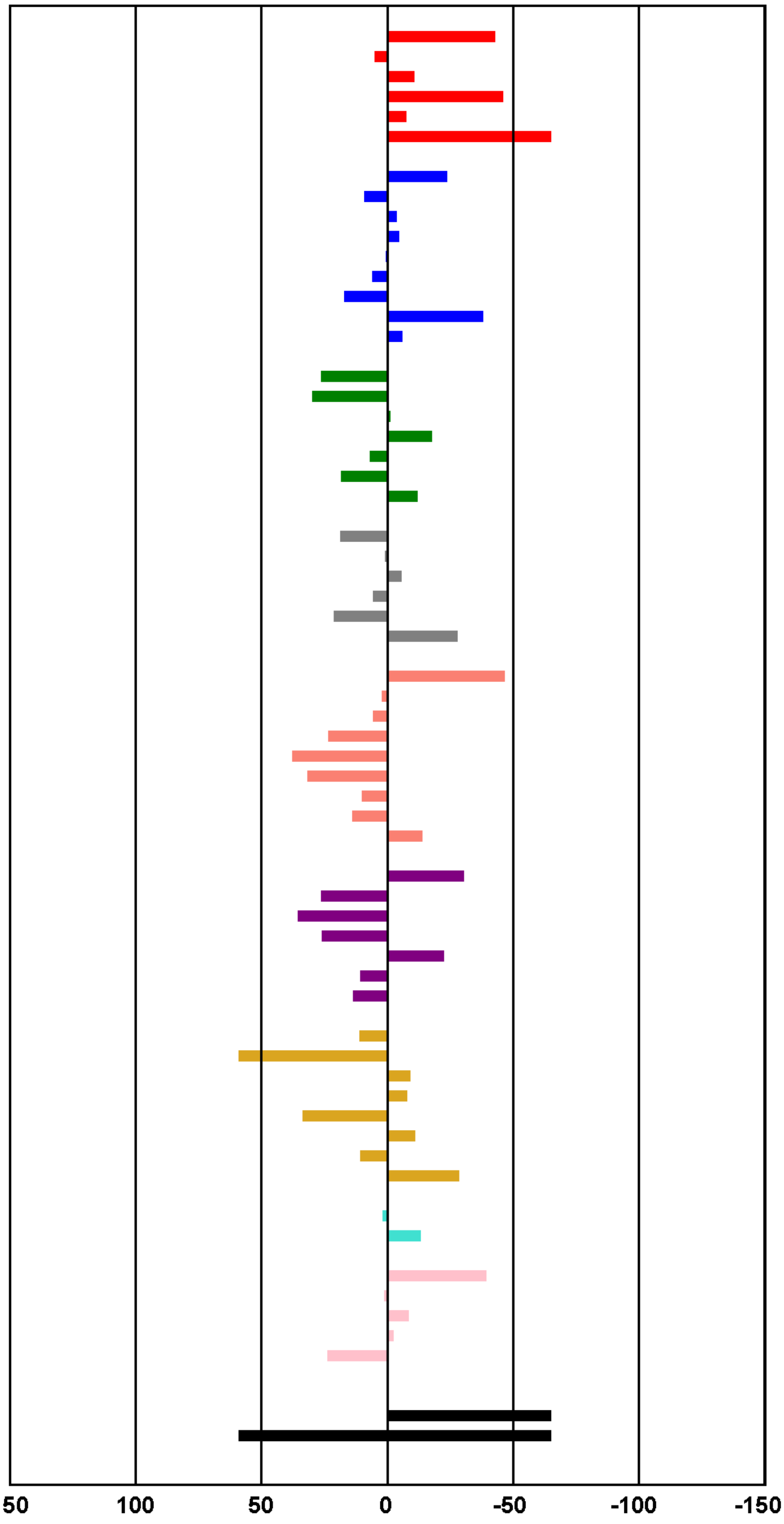

S2 Fig. Anticancer activity (single-dose (10^{-5} M) assay) of the azatetracyclic derivative **1b**.

Developmental Therapeutics Program	NSC: D-834424 / 1	Conc: 1.00E-5 Molar	Test Date: Feb 14, 2022
One Dose Mean Graph	Experiment ID: 2202OS68		Report Date: May 16, 2022

Panel/Cell Line	Growth Percent
Leukemia	
CCRF-CEM	35.61
HL-60(TB)	76.15
K-562	52.29
MOLT-4	22.94
RPMI-8226	54.57
SR	13.63
Non-Small Cell Lung Cancer	
A549/ATCC	40.78
EKVX	43.25
HOP-62	56.75
HOP-92	54.25
NCI-H226	64.18
NCI-H23	69.05
NCI-H322M	89.44
NCI-H460	18.37
NCI-H522	60.21
Colon Cancer	
COLO 205	55.33
HCC-2998	43.54
HCT-116	49.59
HCT-15	34.20
HT29	63.14
KM12	39.74
SW-620	45.24
CNS Cancer	
SF-268	68.05
SF-295	53.97
SF-539	54.86
SNB-19	58.65
SNB-75	66.39
U251	44.26
Melanoma	
LOX IMVI	41.42
MALME-3M	65.60
M14	75.08
MDA-MB-435	79.04
SK-MEL-2	104.06
SK-MEL-28	105.38
SK-MEL-5	61.68
UACC-257	80.09
UACC-62	51.81
Ovarian Cancer	
IGROV1	32.07
OVCAR-3	76.99
OVCAR-4	48.84
OVCAR-5	64.39
OVCAR-8	38.63
NCI/ADR-RES	70.27
SK-OV-3	75.36
Renal Cancer	
786-0	64.64
A498	121.16
ACHN	36.21
CAKI-1	58.34
RXF 393	140.88
SN12C	53.28
TK-10	65.27
UO-31	37.83
Prostate Cancer	
PC-3	56.34
DU-145	52.25
Breast Cancer	
MCF7	5.77
MDA-MB-231/ATCC	67.66
HS 578T	44.34
T-47D	1.99
MDA-MB-468	-8.15
Mean	55.88
Delta	64.03
Range	149.03

Mean Growth Percent - Growth Percent

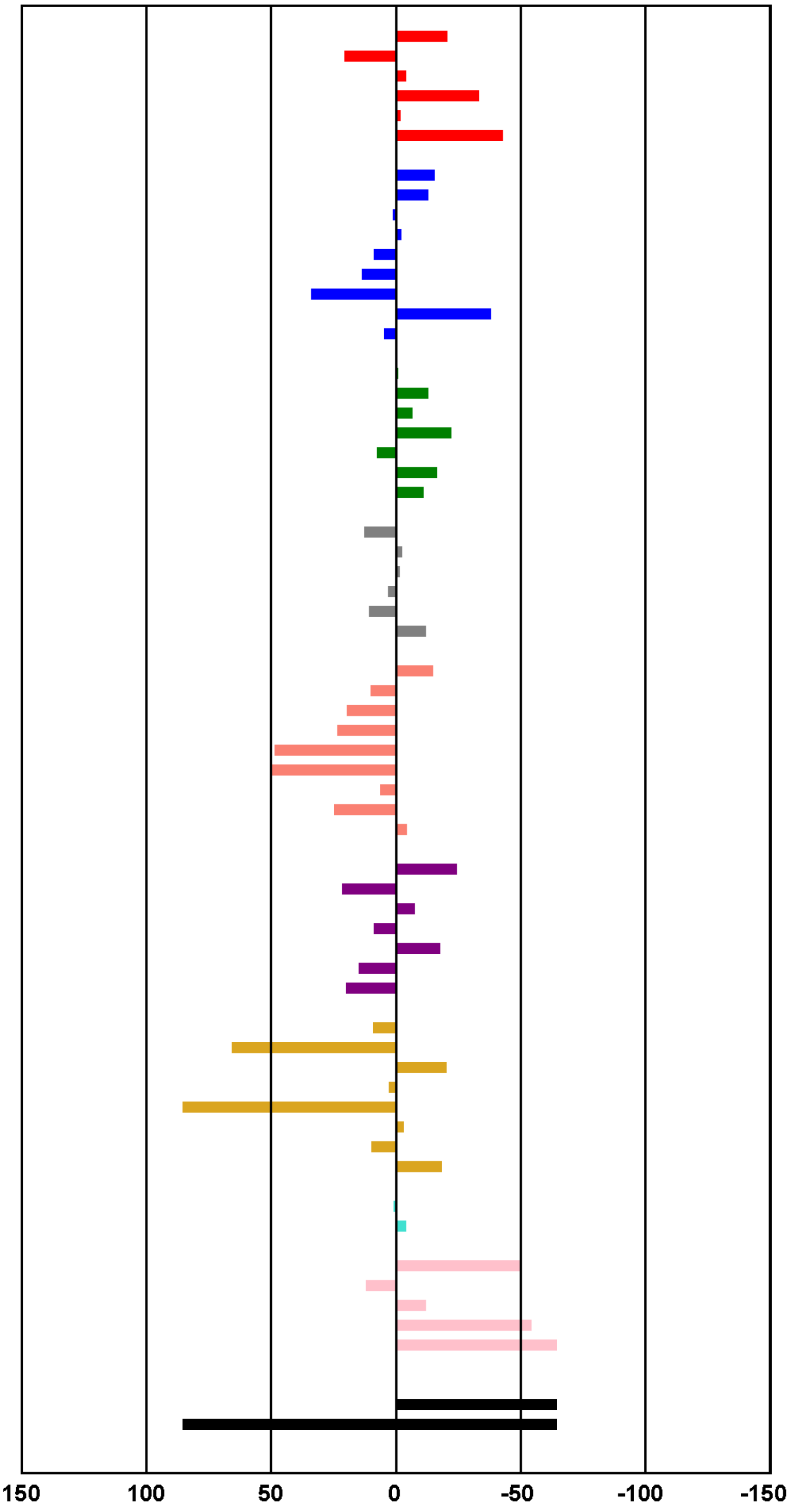

S3 Fig. Anticancer activity (single-dose (10^{-5} M) assay) of the azatetracyclic derivative **2a**.

Developmental Therapeutics Program One Dose Mean Graph	NSC: D-834421 / 1	Conc: 1.00E-5 Molar	Test Date: Feb 14, 2022
	Experiment ID: 2202OS68		Report Date: May 16, 2022

Panel/Cell Line	Growth Percent
**Leukemia**	
CCRF-CEM	72.59
HL-60(TB)	93.79
K-562	64.07
MOLT-4	55.79
RPMI-8226	69.65
SR	56.49
**Non-Small Cell Lung Cancer**	
A549/ATCC	92.83
EKVX	99.50
HOP-62	87.33
HOP-92	111.56
NCI-H226	105.35
NCI-H23	93.50
NCI-H322M	96.14
NCI-H460	109.79
NCI-H522	73.77
**Colon Cancer**	
COLO 205	113.40
HCC-2998	100.53
HCT-116	95.05
HCT-15	69.01
HT29	86.76
KM12	94.89
SW-620	94.77
**CNS Cancer**	
SF-268	111.06
SF-295	103.89
SF-539	98.18
SNB-19	85.53
SNB-75	135.07
U251	70.41
**Melanoma**	
LOX IMVI	44.96
MALME-3M	91.28
M14	98.17
MDA-MB-435	83.93
SK-MEL-2	90.17
SK-MEL-28	99.22
SK-MEL-5	95.74
UACC-257	96.35
UACC-62	72.32
**Ovarian Cancer**	
IGROV1	101.12
OVCAR-3	96.21
OVCAR-4	73.43
OVCAR-5	102.65
OVCAR-8	80.79
NCI/ADR-RES	78.94
SK-OV-3	107.44
**Renal Cancer**	
786-0	97.77
A498	142.65
ACHN	106.34
CAKI-1	97.63
RXF 393	204.87
SN12C	96.39
TK-10	105.26
UO-31	79.61
**Prostate Cancer**	
PC-3	84.22
DU-145	109.60
**Breast Cancer**	
MCF7	70.50
MDA-MB-231/ATCC	101.38
HS 578T	111.02
T-47D	60.69
MDA-MB-468	85.70
Mean	93.34
Delta	48.38
Range	159.91

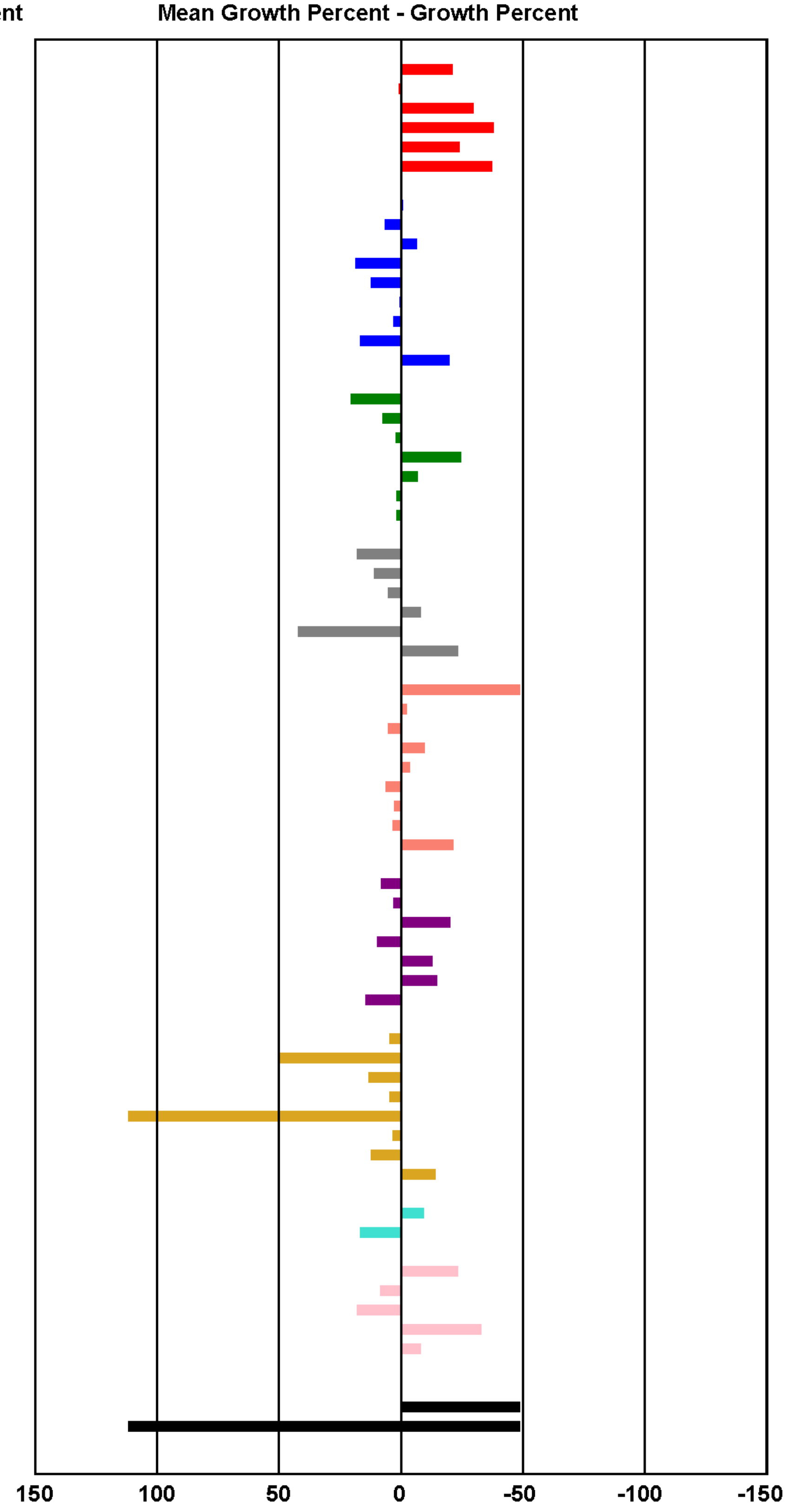

S4 Fig. Anticancer activity (single-dose (10^{-5} M) assay) of the azatetracyclic derivative **3a**.

Developmental Therapeutics Program One Dose Mean Graph	NSC: D-834423 / 1	Conc: 1.00E-5 Molar	Test Date: Feb 14, 2022
	Experiment ID: 2202OS68		Report Date: May 16, 2022

Panel/Cell Line	Growth Percent
Leukemia	
CCRF-CEM	-4.34
HL-60(TB)	39.00
K-562	4.34
MOLT-4	0.19
RPMI-8226	3.26
SR	3.43
Non-Small Cell Lung Cancer	
A549/ATCC	50.54
EKVX	86.51
HOP-62	67.09
HOP-92	53.50
NCI-H226	61.73
NCI-H23	33.44
NCI-H322M	83.45
NCI-H460	17.25
NCI-H522	-55.86
Colon Cancer	
COLO 205	43.17
HCC-2998	67.86
HCT-116	14.03
HCT-15	9.95
HT29	23.99
KM12	91.46
SW-620	9.86
CNS Cancer	
SF-268	69.26
SF-295	78.03
SF-539	17.24
SNB-19	52.10
SNB-75	94.29
U251	23.88
Melanoma	
LOX IMVI	-5.75
MALME-3M	37.92
M14	46.49
MDA-MB-435	10.34
SK-MEL-2	26.89
SK-MEL-28	65.39
SK-MEL-5	24.43
UACC-257	57.02
UACC-62	33.51
Ovarian Cancer	
IGROV1	42.22
OVCAR-3	16.71
OVCAR-4	41.39
OVCAR-5	75.70
OVCAR-8	15.59
NCI/ADR-RES	37.48
SK-OV-3	81.86
Renal Cancer	
786-0	41.92
A498	87.24
ACHN	43.90
CAKI-1	71.31
RXF 393	32.76
SN12C	35.68
TK-10	58.12
UO-31	44.97
Prostate Cancer	
PC-3	47.12
DU-145	71.69
Breast Cancer	
MCF7	6.04
MDA-MB-231/ATCC	33.13
HS 578T	65.59
T-47D	36.50
MDA-MB-468	50.65
Mean	40.21
Delta	96.07
Range	150.15

Mean Growth Percent - Growth Percent

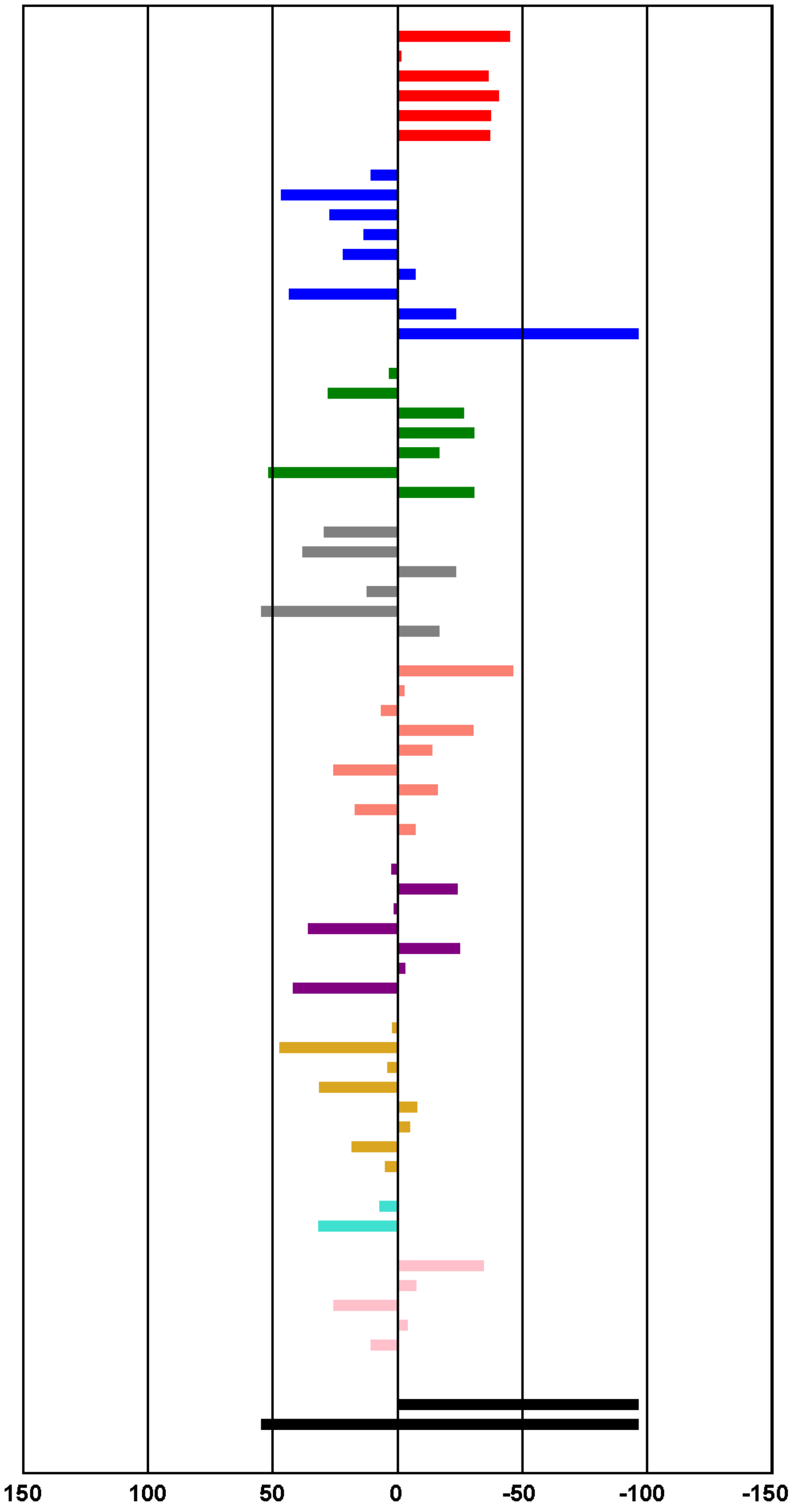

S5 Fig. Anticancer activity (single-dose (10^{-5} M) assay) of the azatetracyclic derivative **3b**.

Developmental Therapeutics Program	NSC: D-834425 / 1	Conc: 1.00E-5 Molar	Test Date: Feb 14, 2022
One Dose Mean Graph	Experiment ID: 2202OS68		Report Date: May 16, 2022

Panel/Cell Line	Growth Percent
Leukemia	
CCRF-CEM	58.92
HL-60(TB)	90.82
K-562	56.89
MOLT-4	65.39
RPMI-8226	71.81
SR	35.75
Non-Small Cell Lung Cancer	
A549/ATCC	100.36
EKVX	104.29
HOP-62	115.19
HOP-92	109.34
NCI-H226	108.71
NCI-H23	98.69
NCI-H322M	100.00
NCI-H460	100.94
NCI-H522	90.14
Colon Cancer	
COLO 205	115.74
HCC-2998	114.29
HCT-116	91.77
HCT-15	82.63
HT29	95.53
KM12	100.35
SW-620	71.85
CNS Cancer	
SF-268	99.99
SF-295	107.71
SF-539	97.47
SNB-19	101.15
SNB-75	102.27
U251	87.95
Melanoma	
LOX IMVI	72.79
MALME-3M	90.42
M14	101.18
MDA-MB-435	96.67
SK-MEL-2	108.22
SK-MEL-28	110.95
SK-MEL-5	99.14
UACC-257	92.39
UACC-62	85.34
Ovarian Cancer	
IGROV1	101.44
OVCAR-3	99.37
OVCAR-4	90.18
OVCAR-5	118.19
OVCAR-8	84.29
NCI/ADR-RES	100.17
SK-OV-3	112.81
Renal Cancer	
786-0	105.00
A498	134.70
ACHN	103.93
CAKI-1	96.13
RXF 393	189.32
SN12C	102.61
TK-10	103.47
UO-31	80.70
Prostate Cancer	
PC-3	100.02
DU-145	105.00
Breast Cancer	
MCF7	62.54
MDA-MB-231/ATCC	100.42
HS 578T	101.95
T-47D	77.30
MDA-MB-468	109.04
Mean	96.81
Delta	61.06
Range	153.57

Mean Growth Percent - Growth Percent

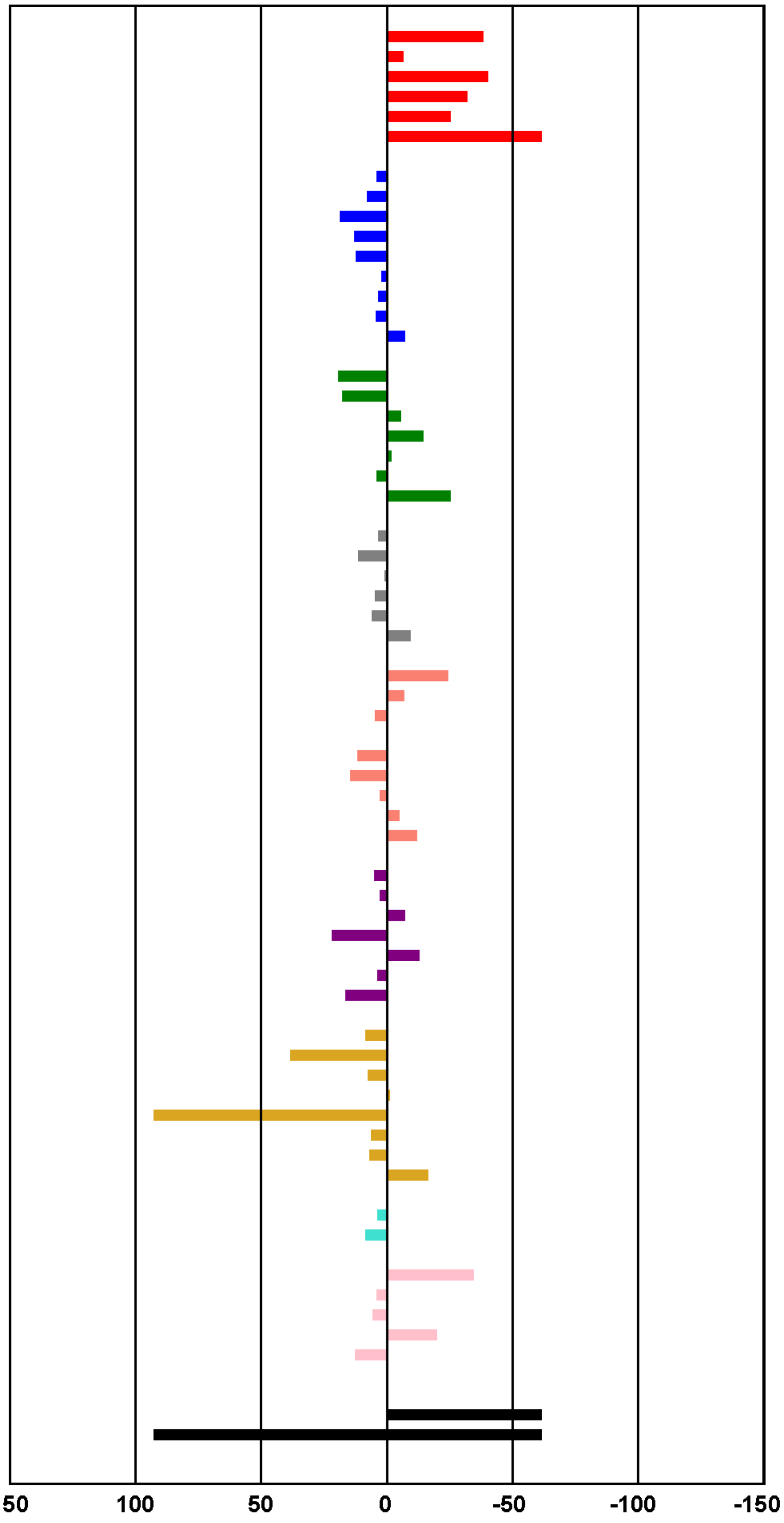

S6 Fig. Anticancer activity (single-dose (10^{-5} M) assay) of the azatetracyclic derivative **4a**.

Developmental Therapeutics Program

One Dose Mean Graph

NSC: D-851948 / 1	Conc: 1.000E-5 M	Test Date: Jan 8, 2024
Experiment ID: 2401HT35		Report Date: Feb 23, 2024

Panel/Cell Line	Growth Percent
**Leukemia**	
CCRF-CEM	-89.27
HL-60(TB)	-98.76
K-562	103.26
MOLT-4	-99.78
RPMI-8226	-87.72
**Non-Small Cell Lung Cancer**	
A549/ATCC	-40.04
EKVX	-44.96
HOP-62	-20.14
HOP-92	-6.75
NCI-H226	-2.49
NCI-H23	-2.76
NCI-H322M	-15.06
NCI-H460	-88.39
NCI-H522	7.37
**Colon Cancer**	
COLO 205	-44.92
HCC-2998	-9.58
HCT-116	23.93
HCT-15	8.23
HT29	33.07
KM12	31.89
SW-620	36.45
**CNS Cancer**	
SF-268	23.97
SF-295	-15.34
SF-539	0.02
SNB-19	28.81
SNB-75	-8.67
U251	25.88
**Melanoma**	
LOX IMVI	29.69
MALME-3M	46.19
M14	29.21
MDA-MB-435	61.27
SK-MEL-2	39.09
SK-MEL-28	52.34
SK-MEL-5	38.37
UACC-257	79.61
UACC-62	33.22
**Ovarian Cancer**	
IGROV1	27.34
OVCAR-3	8.14
OVCAR-4	68.51
OVCAR-8	26.29
NCI/ADR-RES	31.45
SK-OV-3	-61.57
**Renal Cancer**	
786-0	19.61
A498	-47.64
ACHN	-7.12
CAKI-1	23.81
RXF 393	11.00
SN12C	7.40
TK-10	0.04
**Prostate Cancer**	
PC-3	68.79
DU-145	-2.01
**Breast Cancer**	
MCF7	13.98
HS 578T	-19.82
BT-549	27.99
MDA-MB-468	-52.79
Mean	3.65
Delta	103.43
Range	203.04

Mean Growth Percent - Growth Percent

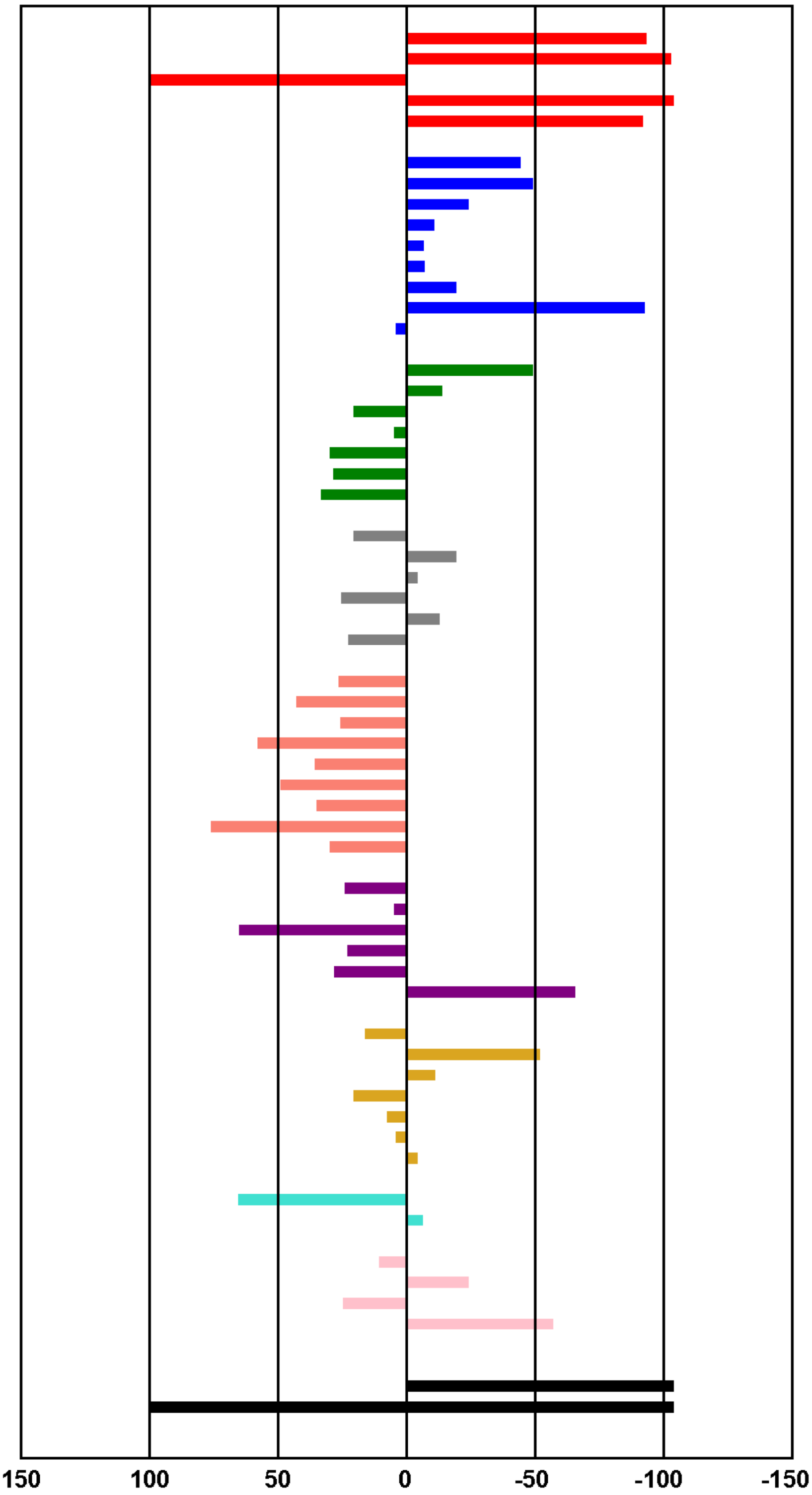

S7 Fig. Anticancer activity (single-dose (10^{-5} M) assay) of the azatetracyclic derivative **5a**.

Developmental Therapeutics Program	NSC: D-852162 / 1	Conc: 1.000E-5 M	Test Date: Apr 22, 2024
One Dose Mean Graph	Experiment ID: 2404HT36		Report Date: Jun 4, 2024

Panel/Cell Line	Growth Percent
Leukemia	
CCRF-CEM	41.02
HL-60(TB)	-38.08
K-562	48.75
MOLT-4	-96.92
RPMI-8226	16.43
SR	-59.19
Non-Small Cell Lung Cancer	
A549/ATCC	49.76
EKVX	10.90
HOP-92	44.00
NCI-H226	-5.11
NCI-H23	45.56
NCI-H322M	104.80
NCI-H460	23.64
NCI-H522	54.57
Colon Cancer	
COLO 205	65.67
HCC-2998	81.57
HCT-116	53.27
HCT-15	68.79
HT29	80.53
KM12	43.08
SW-620	39.36
CNS Cancer	
SF-268	24.93
SF-539	55.75
SNB-19	47.50
SNB-75	28.31
Melanoma	
LOX IMVI	48.24
MALME-3M	54.02
M14	55.35
MDA-MB-435	101.15
SK-MEL-2	80.04
SK-MEL-28	67.89
SK-MEL-5	58.20
UACC-257	79.07
UACC-62	53.43
Ovarian Cancer	
IGROV1	48.88
OVCAR-3	66.95
OVCAR-4	81.81
OVCAR-5	70.75
OVCAR-8	35.74
NCI/ADR-RES	65.25
SK-OV-3	75.76
Renal Cancer	
786-0	45.32
A498	47.55
ACHN	35.68
CAKI-1	48.44
SN12C	30.03
TK-10	39.21
Prostate Cancer	
PC-3	67.45
DU-145	62.38
Breast Cancer	
MCF7	37.63
MDA-MB-231/ATCC	80.45
HS 578T	-12.64
T-47D	1.18
Mean	44.42
Delta	141.34
Range	201.72

Mean Growth Percent - Growth Percent

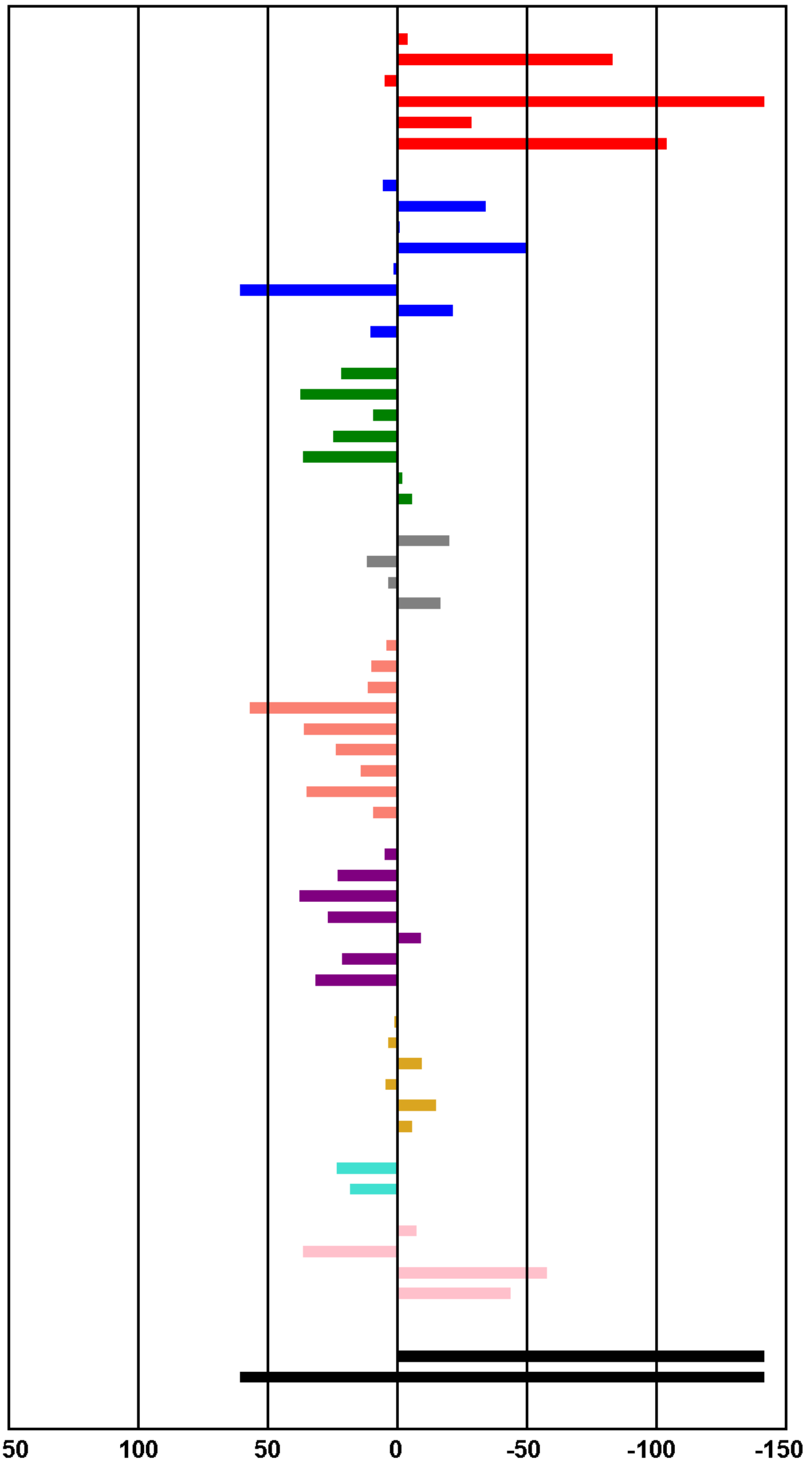

S8 Fig. Anticancer activity (single-dose (10^{-5} M) assay) of the azatetracyclic derivative **5b**.

Developmental Therapeutics Program One Dose Mean Graph	NSC: D-852161 / 1	Conc: 1.000E-5 M	Test Date: Apr 22, 2024
	Experiment ID: 2404HT36		Report Date: Jun 4, 2024

Panel/Cell Line	Growth Percent
Leukemia	
CCRF-CEM	32.86
HL-60(TB)	67.39
K-562	65.36
MOLT-4	12.47
RPMI-8226	45.88
SR	-6.70
Non-Small Cell Lung Cancer	
A549/ATCC	94.04
EKVX	-0.21
HOP-92	85.49
NCI-H226	53.68
NCI-H23	82.68
NCI-H322M	97.98
NCI-H460	83.19
NCI-H522	59.00
Colon Cancer	
COLO 205	110.30
HCC-2998	110.28
HCT-116	81.15
HCT-15	95.37
HT29	87.55
KM12	94.48
SW-620	106.06
CNS Cancer	
SF-268	84.41
SF-539	90.78
SNB-19	81.57
SNB-75	50.95
Melanoma	
LOX IMVI	64.85
MALME-3M	89.92
M14	103.53
MDA-MB-435	113.88
SK-MEL-2	76.09
SK-MEL-28	99.13
SK-MEL-5	42.40
UACC-257	64.75
UACC-62	78.91
Ovarian Cancer	
IGROV1	70.22
OVCAR-3	77.58
OVCAR-4	91.13
OVCAR-5	102.10
OVCAR-8	74.55
NCI/ADR-RES	81.88
SK-OV-3	83.40
Renal Cancer	
786-0	82.56
A498	82.33
ACHN	69.93
CAKI-1	77.90
SN12C	76.83
TK-10	67.62
Prostate Cancer	
PC-3	88.92
DU-145	90.19
Breast Cancer	
MCF7	42.04
MDA-MB-231/ATCC	88.88
HS 578T	39.70
T-47D	52.19
Mean	74.33
Delta	81.03
Range	120.58

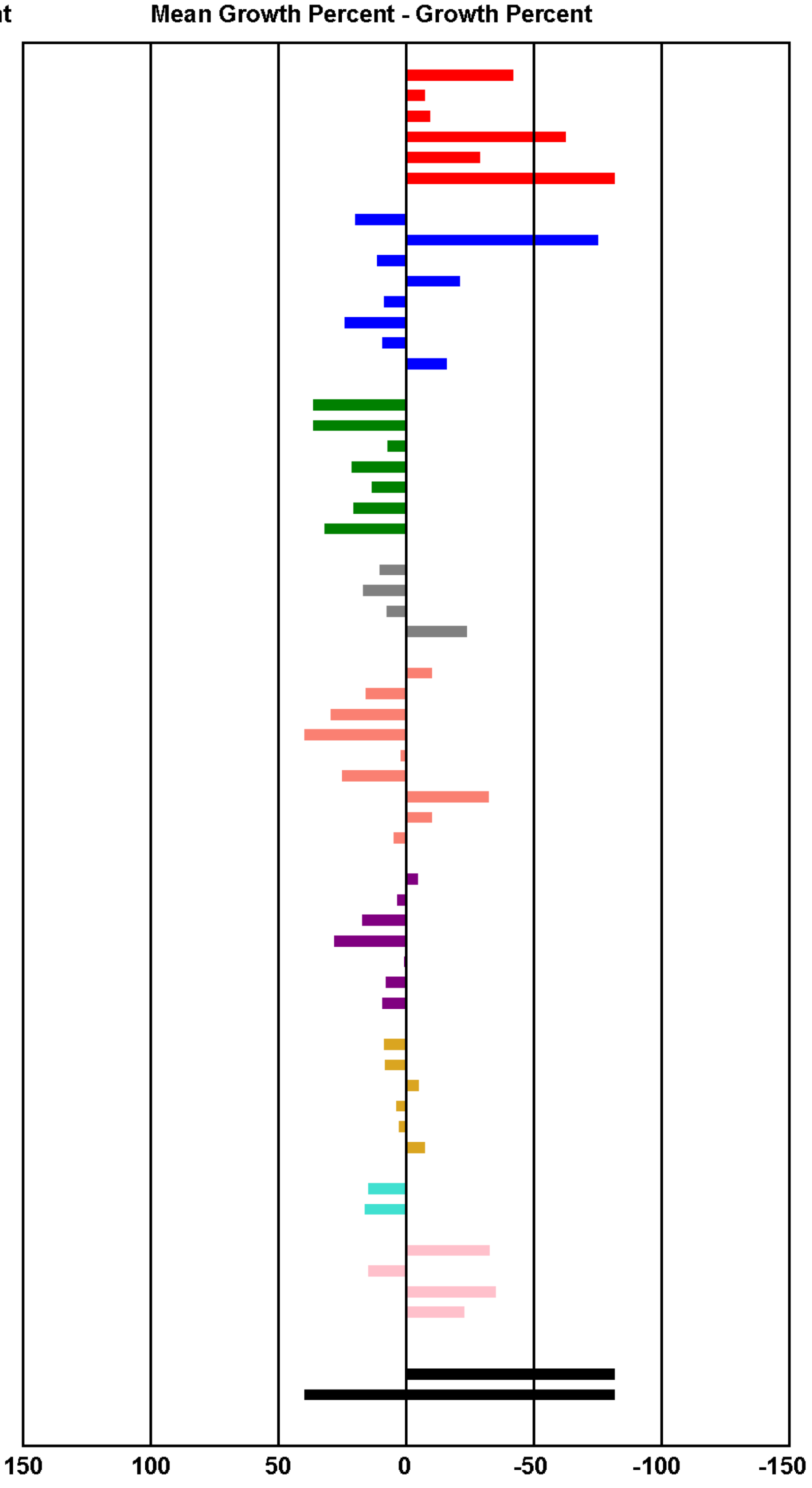

S9 Fig. Anticancer activity (single-dose (10^{-5} M) assay) of the azatetracyclic derivative **6a**.

Developmental Therapeutics Program	NSC: D-852163 / 1	Conc: 1.000E-5 M	Test Date: Apr 22, 2024
One Dose Mean Graph	Experiment ID: 2404HT36		Report Date: Jun 4, 2024

Panel/Cell Line	Growth Percent
Leukemia	
CCRF-CEM	-19.21
HL-60(TB)	34.99
K-562	34.18
MOLT-4	-93.71
RPMI-8226	-30.48
SR	-97.63
Non-Small Cell Lung Cancer	
A549/ATCC	100.41
EKVX	64.59
HOP-92	73.56
NCI-H226	20.33
NCI-H23	65.71
NCI-H322M	79.42
NCI-H460	51.67
NCI-H522	-44.21
Colon Cancer	
COLO 205	90.84
HCC-2998	95.64
HCT-116	51.62
HCT-15	81.62
HT29	76.35
KM12	72.49
SW-620	66.52
CNS Cancer	
SF-268	53.51
SF-539	64.60
SNB-19	70.77
SNB-75	51.79
Melanoma	
LOX IMVI	36.98
MALME-3M	80.39
M14	80.81
MDA-MB-435	90.92
SK-MEL-2	13.25
SK-MEL-28	64.84
SK-MEL-5	22.97
UACC-257	24.67
UACC-62	43.60
Ovarian Cancer	
IGROV1	72.30
OVCAR-3	42.98
OVCAR-4	60.84
OVCAR-5	86.29
OVCAR-8	56.80
NCI/ADR-RES	65.94
SK-OV-3	96.40
Renal Cancer	
786-0	72.96
A498	82.28
ACHN	69.38
CAKI-1	47.36
SN12C	78.10
TK-10	57.94
Prostate Cancer	
PC-3	61.08
DU-145	86.64
Breast Cancer	
MCF7	48.16
MDA-MB-231/ATCC	46.30
HS 578T	24.89
T-47D	58.38
Mean	50.73
Delta	148.36
Range	198.04

Mean Growth Percent - Growth Percent

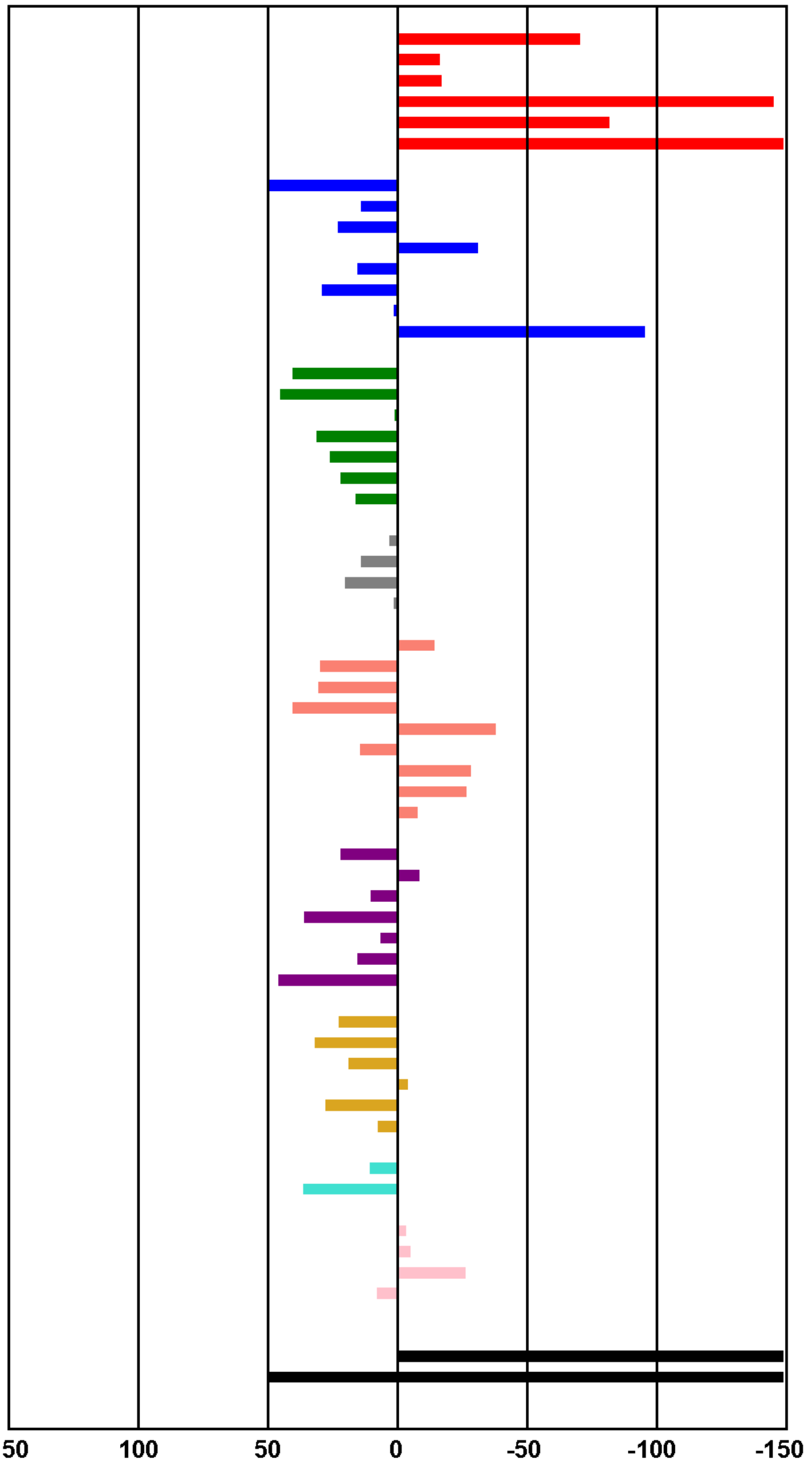

S10 Fig. Anticancer activity (single-dose (10^{-5} M) assay) of the azatetracyclic derivative **6b**.

2. Anticancer activity five-dose assay

National Cancer Institute Developmental Therapeutics Program
In-Vitro Testing Results

NSC : D - 834423 / 1	Experiment ID : 2204NS02	Test Type : 08	Units : Molar
Report Date : September 26, 2023	Test Date : April 18, 2022	QNS :	MC :
COMI : GZ30	Stain Reagent : SRB Dual-Pass Related	SSPL : 0GLI	

			Log10 Concentration												
	Time		Mean Optical Densities					Percent Growth							
Panel/Cell Line	Zero	Ctrl	-8.0	-7.0	-6.0	-5.0	-4.0	-8.0	-7.0	-6.0	-5.0	-4.0	GI50	TGI	LC50
**Leukemia**															
CCRF-CEM	0.418	1.943	1.755	1.824	1.706	0.821	0.428	88	92	84	26	1	3.92E-6	> 1.00E-4	> 1.00E-4
HL-60(TB)	0.517	2.671	2.579	2.636	2.196	1.374	0.636	96	98	78	40	6	5.39E-6	> 1.00E-4	> 1.00E-4
K-562	0.200	2.152	2.138	2.151	1.985	0.988	0.318	99	100	91	40	6	6.48E-6	> 1.00E-4	> 1.00E-4
MOLT-4	0.547	2.710	2.717	2.874	2.550	0.771	0.683	100	108	93	10	6	3.30E-6	> 1.00E-4	> 1.00E-4
RPMI-8226	0.772	2.939	2.943	2.953	2.693	1.732	0.613	100	101	89	44	-21	7.43E-6	4.81E-5	> 1.00E-4
SR	0.195	0.760	0.770	0.799	0.675	0.418	0.385	102	107	85	40	34	5.87E-6	> 1.00E-4	> 1.00E-4
**Non-Small Cell Lung Cancer**															
A549/ATCC	0.383	2.209	2.112	2.117	2.082	1.854	0.962	95	95	93	81	32	4.23E-5	> 1.00E-4	> 1.00E-4
EKVX	0.574	2.003	1.897	1.942	1.835	1.624	1.089	93	96	88	74	36	4.24E-5	> 1.00E-4	> 1.00E-4
HOP-62	0.328	1.150	1.095	1.208	1.223	0.985	0.512	93	107	109	80	22	3.31E-5	> 1.00E-4	> 1.00E-4
HOP-92	1.014	2.006	1.876	1.922	1.833	1.577	1.209	87	92	83	57	20	1.52E-5	> 1.00E-4	> 1.00E-4
NCI-H226	0.759	1.550	1.485	1.488	1.455	1.273	0.983	92	92	88	65	28	2.56E-5	> 1.00E-4	> 1.00E-4
NCI-H23	0.432	1.525	1.503	1.513	1.425	1.031	0.559	98	99	91	55	12	1.29E-5	> 1.00E-4	> 1.00E-4
NCI-H322M	0.884	2.232	2.161	2.187	2.190	2.066	1.815	95	97	97	88	69	> 1.00E-4	> 1.00E-4	> 1.00E-4
NCI-H460	0.369	3.200	3.248	3.235	3.135	2.150	0.694	102	101	98	63	11	1.78E-5	> 1.00E-4	> 1.00E-4
NCI-H522	1.106	2.581	2.374	2.479	2.267	1.398	0.117	86	93	79	20	-89	3.07E-6	1.52E-5	4.35E-5
**Colon Cancer**															
COLO 205	0.556	2.085	2.126	2.128	1.945	1.625	1.108	103	103	91	70	36	3.88E-5	> 1.00E-4	> 1.00E-4
HCC-2998	0.748	2.825	2.589	2.767	2.657	2.362	1.404	89	97	92	78	32	3.99E-5	> 1.00E-4	> 1.00E-4
HCT-116	0.306	2.778	2.663	2.730	2.702	1.590	0.378	95	98	97	52	3	1.10E-5	> 1.00E-4	> 1.00E-4
HCT-15	0.281	2.020	1.982	1.983	1.805	0.977	0.160	98	98	88	40	-43	6.17E-6	3.03E-5	> 1.00E-4
HT29	0.312	2.090	2.004	2.174	1.991	1.511	0.526	95	105	94	67	12	2.06E-5	> 1.00E-4	> 1.00E-4
KM12	0.789	3.356	3.325	3.348	3.349	3.241	2.559	99	100	100	96	69	> 1.00E-4	> 1.00E-4	> 1.00E-4
SW-620	0.366	2.498	2.372	2.313	2.128	1.320	0.316	94	91	83	45	-14	7.26E-6	5.83E-5	> 1.00E-4
**CNS Cancer**															
SF-268	0.944	2.803	2.745	2.725	2.745	2.461	1.366	97	96	97	82	23	3.44E-5	> 1.00E-4	> 1.00E-4
SF-295	0.579	2.304	2.087	2.170	2.112	1.744	1.075	87	92	89	68	29	2.83E-5	> 1.00E-4	> 1.00E-4
SF-539	0.627	2.080	2.000	2.156	2.060	1.423	0.131	94	105	99	55	-79	1.09E-5	2.56E-5	6.05E-5
SNB-19	0.849	2.379	2.289	2.328	2.222	2.115	1.411	94	97	90	83	37	5.15E-5	> 1.00E-4	> 1.00E-4
SNB-75	1.136	2.090	1.901	1.953	1.923	1.805	1.317	80	86	82	70	19	2.47E-5	> 1.00E-4	> 1.00E-4
U251	0.357	1.724	1.620	1.687	1.600	1.227	0.515	92	97	91	64	12	1.83E-5	> 1.00E-4	> 1.00E-4
**Melanoma**															
LOX IMVI	0.289	2.446	2.346	2.402	2.105	0.950	0.141	95	98	84	31	-51	4.35E-6	2.37E-5	9.66E-5
MALME-3M	0.545	1.186	1.118	1.136	1.068	0.810	0.415	89	92	82	41	-24	6.10E-6	4.30E-5	> 1.00E-4
M14	0.492	1.995	1.977	1.987	1.947	1.590	0.890	99	99	97	73	26	3.13E-5	> 1.00E-4	> 1.00E-4
MDA-MB-435	0.645	2.920	2.838	2.882	2.729	2.064	0.702	96	98	92	62	3	1.61E-5	> 1.00E-4	> 1.00E-4
SK-MEL-2	1.313	2.750	2.711	2.750	2.664	2.282	1.289	97	100	94	67	-2	1.79E-5	9.41E-5	> 1.00E-4
SK-MEL-28	0.539	1.566	1.553	1.628	1.512	1.236	0.788	99	106	95	68	24	2.57E-5	> 1.00E-4	> 1.00E-4
SK-MEL-5	0.759	3.048	3.002	3.056	2.970	2.139	0.534	98	100	97	60	-30	1.30E-5	4.68E-5	> 1.00E-4
UACC-257	0.960	2.319	2.143	2.212	2.202	1.960	1.464	87	92	91	74	37	4.42E-5	> 1.00E-4	> 1.00E-4
UACC-62	0.750	2.298	2.240	2.311	2.098	1.584	0.809	96	101	87	54	4	1.19E-5	> 1.00E-4	> 1.00E-4
**Ovarian Cancer**															
IGROV1	0.584	2.281	2.246	2.278	2.281	1.889	1.012	98	100	100	77	25	3.32E-5	> 1.00E-4	> 1.00E-4
OVCAR-3	0.519	1.781	1.752	1.870	1.773	1.310	0.608	98	107	99	63	7	1.69E-5	> 1.00E-4	> 1.00E-4
OVCAR-4	0.825	2.132	2.090	2.141	2.076	1.706	1.149	97	101	96	67	25	2.56E-5	> 1.00E-4	> 1.00E-4
OVCAR-5	0.524	1.907	1.837	1.908	1.783	1.492	0.710	95	100	91	70	13	2.25E-5	> 1.00E-4	> 1.00E-4
OVCAR-8	0.454	2.311	2.235	2.348	2.240	1.561	0.535	96	102	96	60	4	1.49E-5	> 1.00E-4	> 1.00E-4
NCI/ADR-RES	0.413	1.663	1.656	1.713	1.613	1.213	0.509	99	104	96	64	8	1.77E-5	> 1.00E-4	> 1.00E-4
SK-OV-3	0.828	1.676	1.654	1.654	1.679	1.566	1.319	97	97	100	87	58	> 1.00E-4	> 1.00E-4	> 1.00E-4
**Renal Cancer**															
786-0	0.624	2.475	2.306	2.390	2.335	1.868	0.887	91	95	92	67	14	2.11E-5	> 1.00E-4	> 1.00E-4
A498	1.672	2.371	2.315	2.411	2.354	2.170	2.013	92	106	97	71	49	8.81E-5	> 1.00E-4	> 1.00E-4
ACHN	0.464	2.079	2.023	2.078	2.026	1.537	0.770	96	100	97	66	19	2.22E-5	> 1.00E-4	> 1.00E-4
CAKI-1	0.647	2.392	2.252	2.277	2.131	1.694	1.160	92	93	85	60	29	2.12E-5	> 1.00E-4	> 1.00E-4
RXF 393	1.100	1.709	1.640	1.715	1.601	1.160	1.017	89	101	82	10	-8	2.79E-6	3.67E-5	> 1.00E-4
SN12C	0.658	2.532	2.454	2.536	2.439	1.829	0.838	96	100	95	62	10	1.72E-5	> 1.00E-4	> 1.00E-4
TK-10	0.856	1.885	1.788	1.907	2.016	1.920	1.298	91	102	113	103	43	7.63E-5	> 1.00E-4	> 1.00E-4
UO-31	0.830	2.689	2.507	2.518	2.434	2.023	1.358	90	91	86	64	28	2.49E-5	> 1.00E-4	> 1.00E-4
**Prostate Cancer**															
PC-3	0.592	1.971	1.908	1.956	1.813	1.381	0.986	95	99	88	57	29	1.78E-5	> 1.00E-4	> 1.00E-4
DU-145	0.578	2.334	2.341	2.411	2.369	2.118	1.581	100	104	102	88	57	> 1.00E-4	> 1.00E-4	> 1.00E-4
**Breast Cancer**															
MCF7	0.454	2.505	2.361	2.350	2.256	1.082	0.517	93	92	88	31	3	4.58E-6	> 1.00E-4	> 1.00E-4
MDA-MB-231/ATCC	0.462	1.317	1.251	1.326	1.236	0.722	0.349	92	101	90	30	-24	4.72E-6	3.58E-5	> 1.00E-4
HS 578T	1.444	2.504	2.367	2.496	2.488	2.185	1.410	87	99	98	70	-2	1.88E-5	9.27E-5	> 1.00E-4
BT-549	1.180	1.755	1.827	1.811	1.725	1.400	0.885	112	110	95	38	-25	6.18E-6	4.02E-5	> 1.00E-4
T-47D	0.961	2.320	2.104	2.200	2.070	1.665	1.265	84	91	82	52	22	1.15E-5	> 1.00E-4	> 1.00E-4
MDA-MB-468	0.748	1.547	1.466	1.499	1.454	1.227	0.573	90	94	88	60	-23	1.32E-5	5.24E-5	> 1.00E-4

S11 Fig. Anticancer activity (five-dose assay) of the azatetracyclic derivative **3b**.

National Cancer Institute Developmental Therapeutics Program
In-Vitro Testing Results

NSC : D - 851948 / 1	Experiment ID : 2407HT56	Test Type : HTS	Units : Molar
Report Date : September 10, 2024	Test Date : July 22, 2024	QNS :	MC :
COMI : GZ73	Stain Reagent :	SSPL : 0GLI	

			Log10 Concentration												
	Time		Mean Optical Densities					Percent Growth							
Panel/Cell Line	Zero	Ctrl	-8.4	-7.4	-6.4	-5.4	-4.4	-8.4	-7.4	-6.4	-5.4	-4.4	GI50	TGI	LC50
Leukemia															
CCRF-CEM	0.971	5.387	5.830	5.919	5.626	5.866	2.364	110	112	105	111	32	* 2.47E-5	> 4.22E-5	> 4.22E-5
HL-60(TB)	0.502	2.975	4.001	4.200	4.271	3.705	0.319	141	150	152	130	-36	* 1.27E-5	* 2.55E-5	> 4.22E-5
K-562	0.421	5.910	5.403	6.441	5.767	6.101	4.173	91	110	98	104	68	> 4.22E-5	> 4.22E-5	> 4.22E-5
MOLT-4	0.728	3.316	3.868	3.801	3.733	2.954	0.010	121	118	116	85	-99	* 6.58E-6	* 1.23E-5	* 2.30E-5
RPMI-8226	4.586	10.817	10.212	11.787	11.428	10.872	4.656	90	115	110	101	1	* 1.36E-5	> 4.22E-5	> 4.22E-5
SR	0.953	6.285	7.252	7.097	7.298	6.561	0.138	118	115	119	105	-86	* 8.23E-6	* 1.51E-5	* 2.75E-5
Non-Small Cell Lung Cancer															
A549/ATCC	0.392	3.905	3.712	4.014	3.748	0.692	0.261	94	103	96	9	-33	* 1.41E-6	* 6.76E-6	> 4.22E-5
EKVX	5.120	12.740	13.245	13.333	13.136	11.302	4.238	107	108	105	81	-17	* 8.77E-6	* 2.82E-5	> 4.22E-5
HOP-62	1.512	4.663	4.190	4.421	4.628	4.843	4.073	85	92	99	105	82	> 4.22E-5	> 4.22E-5	> 4.22E-5
HOP-92	8.037	10.363	11.039	10.966	11.612	10.916	9.494	129	126	154	124	63	> 4.22E-5	> 4.22E-5	> 4.22E-5
NCI-H226	6.623	16.612	15.148	16.167	15.736	15.166	12.540	85	96	91	85	59	> 4.22E-5	> 4.22E-5	> 4.22E-5
NCI-H23	4.634	13.189	12.496	12.852	12.738	12.158	10.935	92	96	95	88	74	> 4.22E-5	> 4.22E-5	> 4.22E-5
NCI-H322M	6.734	14.167	14.018	15.492	16.057	14.962	5.724	98	118	126	111	-15	* 1.28E-5	* 3.21E-5	> 4.22E-5
NCI-H460	0.969	14.782	15.746	15.797	15.519	6.646	0.440	107	107	105	41	-55	* 3.07E-6	* 1.14E-5	* 3.78E-5
NCI-H522	3.316	8.706	9.441	9.911	10.084	9.327	6.938	114	122	126	111	67	> 4.22E-5	> 4.22E-5	> 4.22E-5
Colon Cancer															
COLO 205	0.835	4.099	4.167	4.380	4.286	2.457	0.533	102	109	106	50	-36	* 4.18E-6	* 1.60E-5	> 4.22E-5
HCC-2998	4.833	13.710	12.454	13.376	13.121	10.253	5.472	86	96	94	61	7	* 6.73E-6	> 4.22E-5	> 4.22E-5
HCT-116	0.373	4.662	4.400	4.681	4.780	4.633	3.449	94	100	103	99	72	> 4.22E-5	> 4.22E-5	> 4.22E-5
HCT-15	1.263	13.246	13.497	11.580	12.427	13.069	8.345	103	86	93	99	59	> 4.22E-5	> 4.22E-5	> 4.22E-5
HT29	0.509	4.988	4.674	5.097	5.038	4.940	4.218	93	102	101	99	83	> 4.22E-5	> 4.22E-5	> 4.22E-5
KM12	0.587	3.832	3.971	4.222	3.904	3.858	2.578	104	112	102	101	61	> 4.22E-5	> 4.22E-5	> 4.22E-5
SW-620	0.580	4.922	5.020	5.216	5.260	4.884	3.333	102	107	108	99	63	> 4.22E-5	> 4.22E-5	> 4.22E-5
CNS Cancer															
SF-268	1.084	3.360	3.113	3.232	3.397	3.304	2.696	89	94	101	98	71	> 4.22E-5	> 4.22E-5	> 4.22E-5
SF-295	1.714	4.386	4.726	4.567	4.411	4.365	3.955	113	107	101	99	84	> 4.22E-5	> 4.22E-5	> 4.22E-5
SF-539	4.105	13.050	12.226	12.498	12.729	13.125	12.421	91	94	96	101	93	> 4.22E-5	> 4.22E-5	> 4.22E-5
SNB-19	1.783	4.860	4.967	5.055	5.316	5.203	4.468	103	106	115	111	87	> 4.22E-5	> 4.22E-5	> 4.22E-5
SNB-75	2.056	3.027	2.918	3.060	3.039	2.817	2.732	89	103	101	78	70	> 4.22E-5	> 4.22E-5	> 4.22E-5
U251	0.623	3.612	3.985	4.005	4.131	4.095	3.300	112	113	117	116	90	> 4.22E-5	> 4.22E-5	> 4.22E-5
Melanoma															
LOX IMVI	0.362	2.599	2.981	2.123	1.797	2.948	1.698	117	79	64	116	60	> 4.22E-5	> 4.22E-5	> 4.22E-5
MALME-3M	2.638	4.463	3.984	4.465	4.428	4.611	4.721	74	100	98	108	115	> 4.22E-5	> 4.22E-5	> 4.22E-5
M14	4.190	13.116	13.114	13.673	13.832	13.520	12.163	100	106	108	105	89	> 4.22E-5	> 4.22E-5	> 4.22E-5
MDA-MB-435	1.129	3.589	3.971	4.256	4.466	4.538	4.232	115	127	136	139	126	> 4.22E-5	> 4.22E-5	> 4.22E-5
SK-MEL-2	2.588	5.812	6.054	5.438	5.604	5.392	5.690	108	88	94	87	96	> 4.22E-5	> 4.22E-5	> 4.22E-5
SK-MEL-28	1.628	5.233	5.031	5.039	5.189	4.949	4.723	94	95	99	92	86	> 4.22E-5	> 4.22E-5	> 4.22E-5
SK-MEL-5	2.807	8.505	9.491	9.086	8.976	8.611	6.330	118	110	108	102	62	> 4.22E-5	> 4.22E-5	> 4.22E-5
UACC-257	4.071	8.926	9.588	9.920	9.747	9.617	9.964	114	121	117	114	121	> 4.22E-5	> 4.22E-5	> 4.22E-5
UACC-62	0.765	3.477	3.210	3.412	3.597	3.458	2.507	90	98	104	99	64	> 4.22E-5	> 4.22E-5	> 4.22E-5
Ovarian Cancer															
IGROV1	1.599	4.262	3.923	4.294	4.380	4.303	3.353	87	101	104	101	66	> 4.22E-5	> 4.22E-5	> 4.22E-5
OVCAR-3	2.879	11.192	11.548	12.389	12.423	12.652	10.350	104	114	115	118	90	> 4.22E-5	> 4.22E-5	> 4.22E-5
OVCAR-4	4.638	8.066	8.011	8.996	8.708	8.607	7.738	98	127	119	116	90	> 4.22E-5	> 4.22E-5	> 4.22E-5
OVCAR-5	5.432	12.252	13.259	13.241	13.294	13.264	11.722	115	114	115	115	92	> 4.22E-5	> 4.22E-5	> 4.22E-5
OVCAR-8	0.988	4.937	4.580	4.781	4.941	4.901	3.504	91	96	100	99	64	> 4.22E-5	> 4.22E-5	> 4.22E-5
NCI/ADR-RES	3.228	11.606	12.254	13.019	13.324	13.272	11.480	108	117	121	120	98	> 4.22E-5	> 4.22E-5	> 4.22E-5
SK-OV-3	2.770	12.910	12.564	12.256	12.472	11.688	3.152	97	94	96	88	4	* 1.19E-5	> 4.22E-5	> 4.22E-5
Renal Cancer															
786-0	1.731	5.205	5.347	5.329	5.335	5.434	4.855	104	104	104	107	90	> 4.22E-5	> 4.22E-5	> 4.22E-5
A498	2.609	9.283	9.445	10.066	10.461	6.774	1.724	102	112	118	63	-34	* 5.71E-6	* 1.88E-5	> 4.22E-5
ACHN	1.003	4.530	4.796	4.596	4.770	4.528	2.824	108	102	107	100	52	> 4.22E-5	> 4.22E-5	> 4.22E-5
CAKI-1	1.015	4.800	4.972	5.039	4.880	4.543	2.548	105	106	102	93	40	* 2.78E-5	> 4.22E-5	> 4.22E-5
RXF 393	2.078	3.619	3.871	3.750	3.763	3.676	3.127	116	108	109	103	68	> 4.22E-5	> 4.22E-5	> 4.22E-5
SN12C	0.660	2.803	2.677	2.646	2.743	2.554	1.586	94	93	97	88	43	* 2.98E-5	> 4.22E-5	> 4.22E-5
TK-10	7.459	17.825	18.541	18.139	18.084	17.636	12.796	107	103	103	98	52	> 4.22E-5	> 4.22E-5	> 4.22E-5
UO-31	1.590	6.128	6.089	6.019	5.958	5.812	4.423	99	98	96	93	62	> 4.22E-5	> 4.22E-5	> 4.22E-5
Prostate Cancer															
PC-3	4.255	11.961	12.209	13.142	13.401	13.154	12.060	103	115	119	115	101	> 4.22E-5	> 4.22E-5	> 4.22E-5
DU-145	0.789	3.882	4.096	4.173	4.280	4.305	1.766	107	109	113	114	32	* 2.53E-5	> 4.22E-5	> 4.22E-5
Breast Cancer															
MCF7	0.974	9.173	9.505	8.698	9.927	8.514	4.023	104	94	109	92	37	* 2.45E-5	> 4.22E-5	> 4.22E-5
MDA-MB-231/ATCC	4.073	9.187	10.152	10.197	10.380	9.752	9.392	119	120	123	111	104	> 4.22E-5	> 4.22E-5	> 4.22E-5
HS 578T	2.251	4.235	4.014	4.243	4.297	4.080	3.398	89	100	103	92	58	> 4.22E-5	> 4.22E-5	> 4.22E-5
BT-549	6.928	12.811	12.013	13.255	12.833	13.007	11.318	87	108	101	104	75	> 4.22E-5	> 4.22E-5	> 4.22E-5
T-47D	6.839	11.992	12.134	11.901	12.479	12.047	7.881	103	98	109	101	20	* 1.81E-5	> 4.22E-5	> 4.22E-5
MDA-MB-468	5.295	9.492	9.292	9.587	9.294	8.664	2.730	95	102	95	80	-48	* 7.25E-6	* 1.78E-5	> 4.22E-5

S12 Fig. Anticancer activity (five-dose assay) of the azatetracyclic derivative **5a**.

3. Comparison of the anticancer activity of compounds 3b/ CC260 and 5a/ dexrazoxane respectively

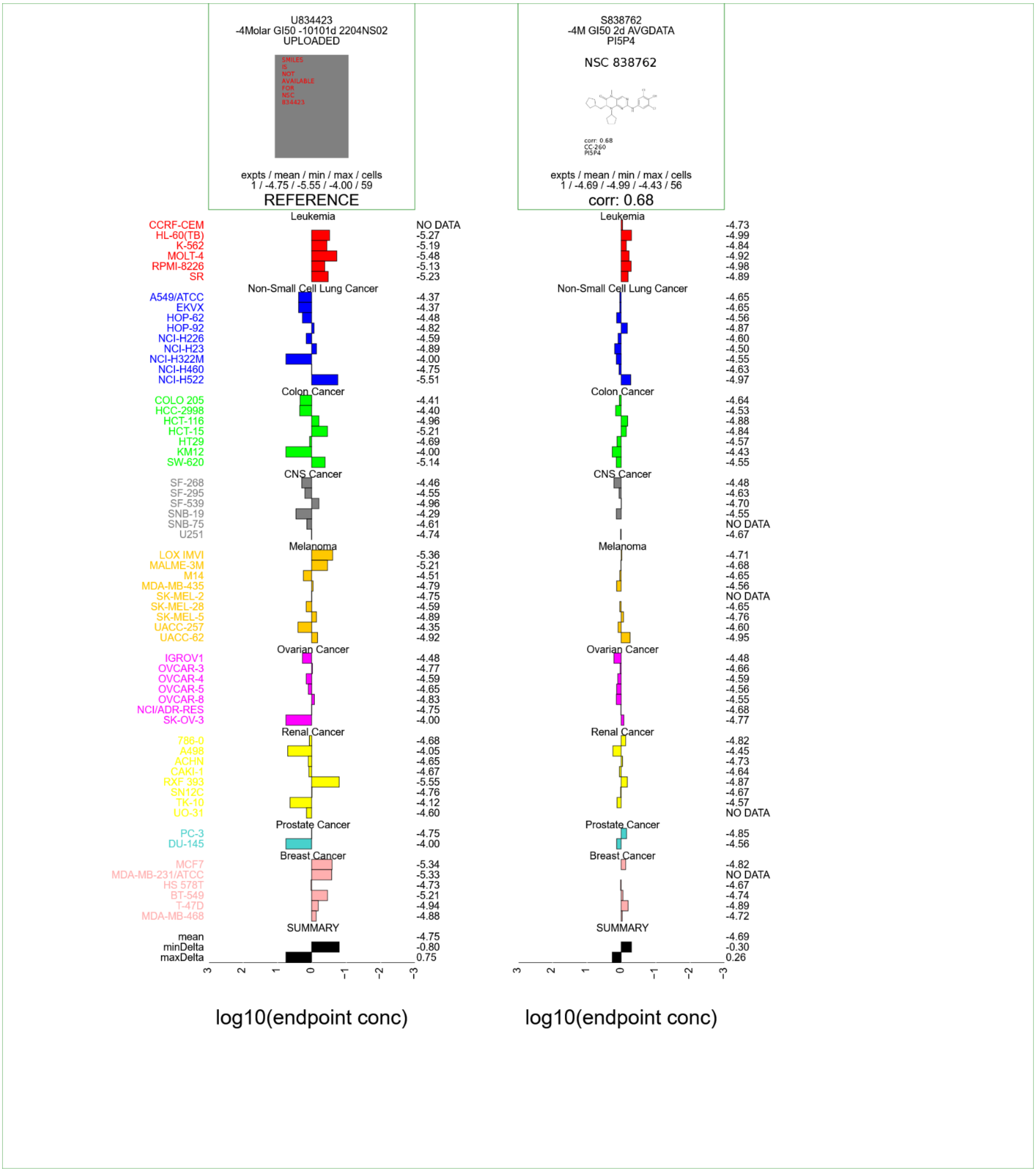

S13 Fig. Comparison of mean graphs of compounds **3b** and **CC260**.

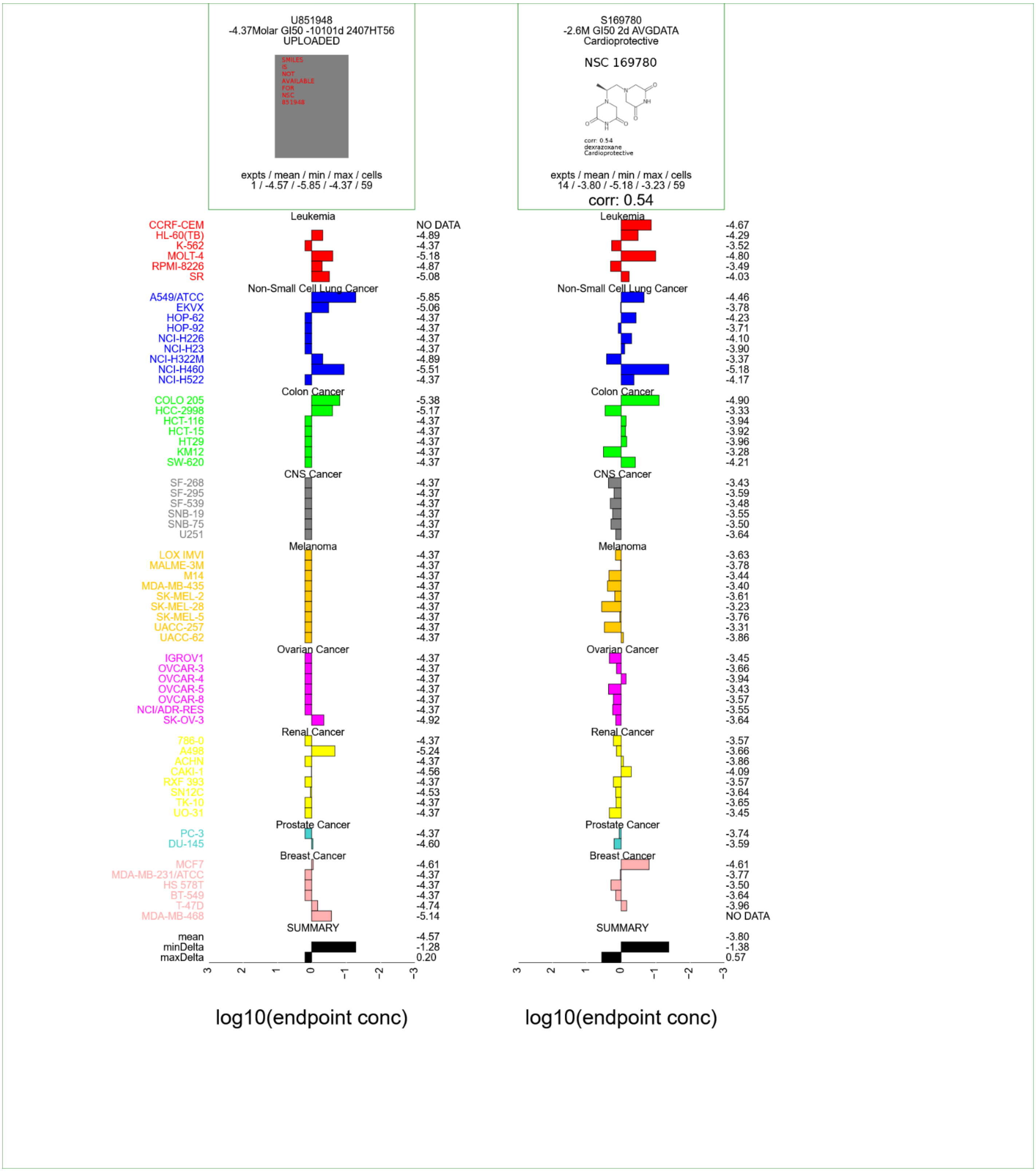

S14 Fig. Comparison of mean graphs of compounds **5a** and **dexrazoxane**.
